# The Importance of Implementation Strategy in Scaling Up Xpert MTB/RIF for Diagnosis of Tuberculosis in the Indian Health-Care System: A Transmission Model

**DOI:** 10.1371/journal.pmed.1001674

**Published:** 2014-07-15

**Authors:** Henrik Salje, Jason R. Andrews, Sarang Deo, Srinath Satyanarayana, Amanda Y. Sun, Madhukar Pai, David W. Dowdy

**Affiliations:** 1 Department of Epidemiology, Johns Hopkins Bloomberg School of Public Health, Baltimore, Maryland, United States of America; 2 Division of Infectious Diseases, Massachusetts General Hospital, Boston, Massachusetts, United States of America; 3 Indian School of Business, Hyderabad, India; 4 Department of Epidemiology and Biostatistics, McGill University, Montreal, Quebec, Canada; 5 McGill International TB Centre, McGill University Health Centre, Montreal, Quebec, Canada; 6 Johns Hopkins School of Medicine, Baltimore, Maryland, United States of America; 7 Montreal Chest Institute, McGill University Health Centre, Montreal, Quebec, Canada; 8 Center for Tuberculosis Research, Johns Hopkins University, Baltimore, Maryland, United States of America; Harvard School of Public Health, United States of America

## Abstract

**Background:**

India has announced a goal of universal access to quality tuberculosis (TB) diagnosis and treatment. A number of novel diagnostics could help meet this important goal. The rollout of one such diagnostic, Xpert MTB/RIF (Xpert) is being considered, but if Xpert is used mainly for people with HIV or high risk of multidrug-resistant TB (MDR-TB) in the public sector, population-level impact may be limited.

**Methods and Findings:**

We developed a model of TB transmission, care-seeking behavior, and diagnostic/treatment practices in India and explored the impact of six different rollout strategies. Providing Xpert to 40% of public-sector patients with HIV or prior TB treatment (similar to current national strategy) reduced TB incidence by 0.2% (95% uncertainty range [UR]: −1.4%, 1.7%) and MDR-TB incidence by 2.4% (95% UR: −5.2%, 9.1%) relative to existing practice but required 2,500 additional MDR-TB treatments and 60 four-module GeneXpert systems at maximum capacity. Further including 20% of unselected symptomatic individuals in the public sector required 700 systems and reduced incidence by 2.1% (95% UR: 0.5%, 3.9%); a similar approach involving qualified private providers (providers who have received at least some training in allopathic or non-allopathic medicine) reduced incidence by 6.0% (95% UR: 3.9%, 7.9%) with similar resource outlay, but only if high treatment success was assured. Engaging 20% of all private-sector providers (qualified and informal [providers with no formal medical training]) had the greatest impact (14.1% reduction, 95% UR: 10.6%, 16.9%), but required >2,200 systems and reliable treatment referral. Improving referrals from informal providers for smear-based diagnosis in the public sector (without Xpert rollout) had substantially greater impact (6.3% reduction) than Xpert scale-up within the public sector. These findings are subject to substantial uncertainty regarding private-sector treatment patterns, patient care-seeking behavior, symptoms, and infectiousness over time; these uncertainties should be addressed by future research.

**Conclusions:**

The impact of new diagnostics for TB control in India depends on implementation within the complex, fragmented health-care system. Transformative strategies will require private/informal-sector engagement, adequate referral systems, improved treatment quality, and substantial resources.

*Please see later in the article for the Editors' Summary*

## Abstract 2

Using a modelling approach, David Dowdy and colleagues investigate how different implementation strategies for Xpert MTB/RIF within the complex, fragmented healthcare system of India may affect tuberculosis control.

*Please see later in the article for the Editors' Summary*

## Editors' Summary

**Background:**

Tuberculosis—a contagious bacterial disease that usually infects the lungs—is a global public health problem. Each year, about 8.7 million people develop active tuberculosis and about 1.4 million people die from the disease. *Mycobacterium tuberculosis*, the bacterium that causes tuberculosis, is spread in airborne droplets when people with active disease cough or sneeze. The characteristic symptoms of tuberculosis are a persistent cough, fever, weight loss, and night sweats. Diagnostic tests for tuberculosis include sputum smear microscopy (microscopic analysis of mucus coughed up from the lungs), the growth of *M. tuberculosis* from sputum samples, and new molecular tests (for example, the automated Xpert MTB/RIF test) that rapidly and accurately detect *M. tuberculosis* in patient samples and determine its resistance to certain antibiotics. Tuberculosis can be cured by taking several antibiotics daily for at least six months, although the recent emergence of multidrug-resistant (MDR) tuberculosis is making the disease increasingly hard to treat.

**Why Was This Study Done?:**

About 25% of all tuberculosis cases occur in India. Most people in India with underlying tuberculosis initially seek care for cough from the private health-care sector, which comprises informal providers with no formal medical training and providers with some training in mainstream or alternative medicine. Private providers rarely investigate for tuberculosis, and patients often move between providers, with long diagnostic delays. The public sector ultimately diagnoses and treats more than half of tuberculosis cases. However, the public sector relies on sputum smear microscopy, which misses half of cases, and the full diagnostic process from symptom onset to treatment initiation can take several months, during which time individuals remain infectious. Could the rollout of molecular diagnostic tests improve tuberculosis control in India? The Indian Revised National Tuberculosis Control Programme (RNTCP) is currently introducing the Xpert MTB/RIF test (Xpert) as a rapid method for drug susceptibility testing in the public sector in people at high risk of MDR tuberculosis, but is this the most effective rollout strategy? Here, the researchers use a mathematical transmission model to investigate the likely effects of the rollout of Xpert in India using different implementation strategies.

**What Did the Researchers Do and Find?:**

The researchers explored the impact of several rollout strategies on the incidence of tuberculosis (the number of new cases of tuberculosis in the population per year) by developing a mathematical model of tuberculosis transmission, care-seeking behavior, and diagnostic/treatment practices in India. Compared to a baseline scenario of no improved diagnostic testing, provision of Xpert to 40% of public-sector patients at high risk of MDR tuberculosis (scenario 1, the current national strategy) reduced the incidence of tuberculosis by 0.2% and the incidence of MDR tuberculosis by 2.4%. Implementation of this strategy required 2,500 additional courses of MDR tuberculosis treatment and the continuous use of 60 Xpert machines, about half the machines procured in India during 2013. A scenario that added access to Xpert for 20% of all individuals with tuberculosis symptoms seeking diagnosis in the public sector and 20% of individuals seeking care from qualified private practitioners to scenario 1 reduced the incidence of tuberculosis by 14.1% compared to the baseline scenario but required more than 2,200 Xpert machines and reliable treatment referral. Notably, a scenario that encouraged informal providers to refer suspected tuberculosis cases to the public sector for smear-based diagnosis (no Xpert rollout) had a greater impact on the incidence of tuberculosis than Xpert scale-up within the public sector.

**What Do These Findings Mean?:**

These findings are subject to considerable uncertainty because of the assumptions made in the transmission model about private-sector treatment patterns, patient care-seeking behavior, and infectiousness, and the quality of the data fed into the model. Nevertheless, these findings suggest that the rollout of Xpert (or other new diagnostic methods with similar characteristics) could substantially reduce the burden of tuberculosis due to poor diagnosis in India. Importantly, these findings highlight how the impact of Xpert rollout relies not only on the accuracy of the test but also on the behavior of patients and providers, the level of access to new tools, and the availability of treatment following diagnosis. Thus, to ensure that new diagnostic methods have the maximum impact on tuberculosis in India, it is necessary to engage the whole private health-care sector and to provide adequate referral systems, improved treatment quality, and increased resources across all health-care sectors.

**Additional Information:**

Please access these websites via the online version of this summary at http://dx.doi.org/10.1371/journal.pmed.1001674.

- The World Health Organization (WHO) provides information (in several languages) on all aspects of tuberculosis, including general information on tuberculosis diagnostics and specific information on the roll out of the Xpert MTB/RIF test; further information about WHO's endorsement of Xpert MTB/RIF is included in a Strategic and Technical Advisory Group for Tuberculosis report; the Global Tuberculosis Report 2013 provides information about tuberculosis around the world, including in India
- The Stop TB Partnership is working towards tuberculosis elimination and provides patient stories about tuberculosis (in English and Spanish); the Tuberculosis Vaccine Initiative (a not-for-profit organization) also provides personal stories about tuberculosis
- The US Centers for Disease Control and Prevention has information about tuberculosis and its diagnosis (in English and Spanish)
- The US National Institute of Allergy and Infectious Diseases also has detailed information on all aspects of tuberculosis
- TBC India provides information about tuberculosis control in India, including information on the RNTCP
- The Initiative for Promoting Affordable and Quality TB Tests promotes WHO-endorsed TB tests in India
- MedlinePlus has links to further information about tuberculosis (in English and Spanish)

## Introduction

Despite being a largely curable disease, tuberculosis (TB) causes 8.7 million new cases and 1.4 million deaths per year, over 25% of which—including cases caused by “totally drug-resistant” strains—occur in India [1],[2]. Over the last 15 y, the Revised National Tuberculosis Control Programme (RNTCP) has made great strides in improving TB control efforts in the public sector. However, TB incidence in the country continues to be stubbornly high [1]. In part, TB control efforts in India have been hampered by inadequate and inconsistent diagnostic and treatment practices [1]–[6].

India has a complex health-care system with a wide range of different informal, private, and public providers. In India, the large private sector [7] diagnoses nearly half of all TB cases [8],[9]. This sector consists of individuals who provide medical care outside of the public system and can be broadly divided into informal providers with no formal medical training (who generally provide very low levels of care [4],[10],[11]) and those who have received at least some training in allopathic or non-allopathic (e.g., Ayurveda, Unani, homeopathic) medicine [8],[12]. Patients select providers based on a number of factors, including convenience, cost, and trust; relative to private care, public-sector care may be free but is often less trusted and less convenient [13]. Furthermore, while the RNTCP may be widely recognized, most people with underlying TB initially seek care for cough, not “presumed TB.” They therefore generally first visit nearby informal providers, including chemists, and later more qualified practitioners [12], often seeing several providers [14] before diagnosis. Public-sector diagnosis is often seen as a last resort for those with no money, no other options, or symptoms sufficiently persistent and severe to suggest TB as a likely diagnosis. Ultimately, over half of patients are diagnosed in the public sector [8],[9]. The full diagnostic process from symptom onset to initiation of treatment can take months, during which individuals remain infectious [14]. This fragmented system of care-seeking has spurred efforts to encourage private providers to adhere to Standards for TB Care in India and refer individuals with suspected TB to the public sector [15],[16].

Currently, however, the public sector relies on sputum smear microscopy that misses half of all cases [17], and while culture is available on a limited basis, culture results rarely influence clinical decision-making [18]. The private sector often uses diagnostic tests with even worse performance [6]. Serological, antibody-based tests were popular until their ban in 2012 on the basis of high cost (often $30 or more) and poor accuracy [19]. Subsequently, concern has been raised about the growing use of interferon-gamma release assays (IGRAs) for active TB diagnosis [5],[6],[20]. Both antibody and IGRA tests are discouraged by the current Standards for TB Care in India [15]. Testing for multidrug-resistant TB (MDR-TB) is rarely undertaken in either the public or private sector [1],[21].

Xpert MTB/RIF (Xpert, Cepheid Inc, Sunnyvale, CA, USA) is a novel molecular test for active TB that uses semi-automated PCR to amplify mycobacterial DNA [22],[23]. It has a higher sensitivity than sputum smear microscopy, and, in addition, through the use of specific primers it can detect mutations associated with rifampin resistance. Whereas TB culture can take months to provide these results, Xpert can be performed in 90 min [22]. A demonstration study—including testing in India—found that Xpert could be performed with high sensitivity in decentralized settings [24], a feasibility study showed that obstacles such as high temperature and error rates could be overcome in India [25], and prior modeling analyses in southern Africa have suggested that Xpert rollout could have important epidemiological impact [26].

Currently, the RNTCP is mainly implementing Xpert not for broad high-sensitivity diagnosis of TB but rather primarily as a rapid drug susceptibility testing (DST) method in adults and children with HIV or high risk of MDR-TB [15],[16],[25],[27]. While there is some effort to expand access to the private sector via public–private mix models [27], the central goal is currently to improve DST capacity in the public sector. The comparative population-level impact of various potential rollout strategies in the public versus private sectors remains unclear, especially as this impact depends not just on test accuracy, but also on the appropriate placement of the test—and linkage to appropriate treatment—early in the diagnostic pathway. To better understand the potential epidemiological impact and resource requirements for strategies in which Xpert is scaled up across different health-care sectors, we constructed a transmission model of TB that incorporates provider and patient behavioral patterns within the Indian health-care system.

## Methods

### Model Structure: TB Diagnosis, Treatment, and Care-Seeking

**Figure 1:**
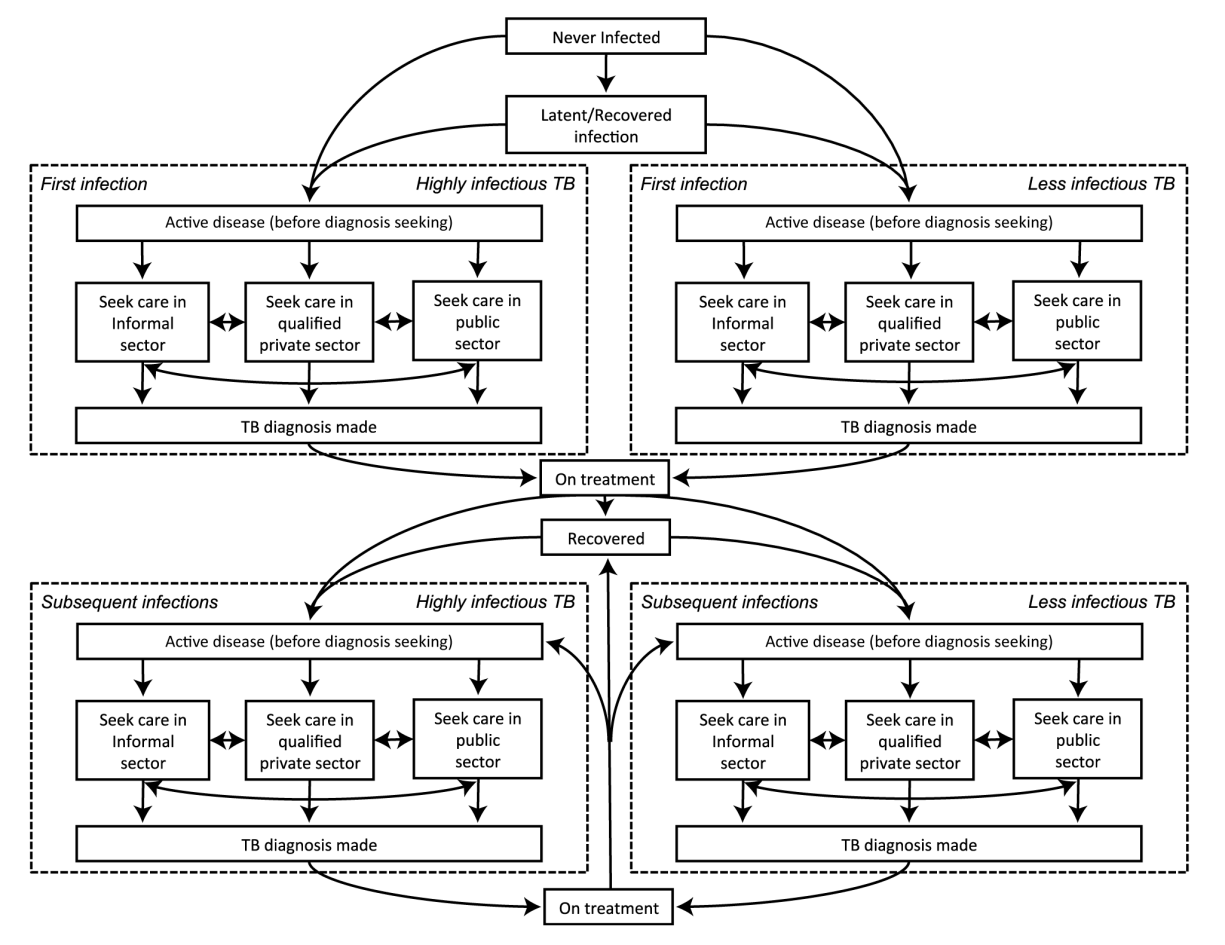
Model schematic. Diagram of the compartments in the model. Not shown, but present in the model, are parallel structures by (a) HIV status and (b) MDR-TB status.

We developed a compartmental model that uses ordinary differential equations to describe the current TB epidemic and health-care system in India (Figures 1 and S1). Regarding TB natural history, this model expands on other published models of TB by incorporating a “before diagnosis-seeking stage” (partially infectious but not seeking diagnosis for any symptoms [28],[29]) and a diagnosis-seeking phase (symptoms sufficiently severe to prompt care-seeking). During diagnosis-seeking, TB is categorized as either highly infectious (diagnosable by sputum smear) or less infectious (not diagnosable by sputum smear). Rates of seeking diagnosis are based on estimates from the literature [12], with each diagnostic attempt occurring in the informal sector (providers with no formal medical training), the qualified private sector (providers who have received at least some training in allopathic or non-allopathic medicine), or the public sector (medically trained providers in the national health-care system) (Table 1). The probability of successful diagnosis and treatment differs according to sector, and both the speed of diagnosis and probability of loss to follow-up before treatment differ across tests. After unsuccessful diagnostic attempts, patients continue seeking diagnosis, with the sector of the subsequent diagnostic attempt conditional on the preceding attempt. Thus, individuals tend to progress from seeking care initially in the informal sector, then the qualified private sector, and finally the public sector as repeated diagnostic attempts fail. Since the diagnosis of pediatric and extrapulmonary TB is generally distinct from that of adult pulmonary TB, we consider only the latter. Two authors independently checked and ran the code to minimize errors. Model parameters are shown in Table 2. A detailed description of the model compartments and equations is set out in Tables S1 and S2 and Texts S1 and S2.

**Table 1:** Movement between health-care providers.

Care-Seeking Behavior	Informal	Qualified Private	Public
**Initial provider approached**	69%	31%	0%
**Subsequent provider visited**			
Previous provider informal	48%	49%	3%
Previous provider qualified private	3%	36%	61%
Previous provider public	0%	0%	100%

Where TB infected individuals initially go to seek diagnosis and the location of subsequent visits [12].

**Table 2:** Model parameters.

Parameter	Value	Sensitivity Range	Source
**Transmissibility, highly infectious TB**	12.0 y^−1^	—a	Fitted value
**Adult mortality rate**	0.02 y^−1^	0.015–0.025	[8],[45]
**HIV adult mortality rate**	0.05 y^−1^	0.025–0.1	[28],[29],[46]
**TB mortality rate, HIV-negative individuals, highly infectious TB**	0.22 y^−1^	0.3–0.5	[12],[47]
**Relative mortality in highly infectious versus less infectious TB, HIV-negative individuals** b	0.29	0.20–0.35	[21],[30],[47]
**TB mortality rate, HIV-positive individuals**	1 y^−1^	0.75–1.25	[38],[48]–[50]
**Proportion of cases that are highly infectious**			
HIV-negative individuals	0.65	0.5–0.8^c^	[17],[49]
HIV-positive individuals	0.35	0.26–0.44^c^	[1],[17],[51]
**Proportion of infections that progress rapidly to active TB**			
HIV-negative individuals	0.14	0.05–0.2^c^^,^ d	[1],[52]
HIV-positive individuals	0.4	0.2–0.8^c^	[1],[53]
**Relative transmission capability of less infectious TB**	0.22	0.17–0.28	[8],[54]
**Lost to follow-up between initial presentation and treatment initiation**		—	[35],[36]
Xpert, smear	0.15	0.05–0.25	
Other diagnostics	0.25	0.13–0.5^c^	
**Proportion of patients with drug-susceptible TB who develop MDR-TB during treatment**	0.008	0.006–0.01	[1],[55]
**Endogenous activation of latent TB, per year**			[56]
HIV-negative individuals	0.001	0.0007–0.0012^c^^,^ d	
HIV-positive individuals	0.023	0.017–0.029^c^^,^ d	
**Protective factor from previous infection, HIV-negative individuals only**	0.6	0.2–0.8	[52]
**Time infectious before symptoms**	4.1 mo	0–6 mo	Fitted value
**Time from symptoms to seek diagnosis**	4.8 mo	1–6 mo	[12]
**Initial sector visited by TB suspect**			[12]
Informal sector	0.69	0.61–0.77	
Qualified private sector	0.31	0.23–0.39	
Public sector	0.00	0–0.2	
**Time to seek second opinion**	1.9 mo	1.5–2.4 mo	[12]
**Site of next diagnostic attempt (informal/qualified private/public)**		—e	[12]
Current diagnostic attempt: informal sector	0.48/0.49/0.03	—	
Current diagnostic attempt: qualified private sector	0.03/0.36/0.61	—	
Current diagnostic attempt: public sector	0/0/1	—	
**Probability of being diagnosed correctly, primary infection, highly infectious TB** f			
Informal sector (without Xpert)	0	Unchanged	Model assumption
Qualified private sector (without Xpert)	0.38	0.27–0.45	Fitted value
Public sector (without Xpert)	0.98	0.85–1	Model assumption
Xpert	0.98	0.85–1	[22]
**Probability of being diagnosed correctly, primary infection, less infectious TB** f			
Informal sector (without Xpert)	0	Unchanged	Model assumption
Qualified private sector (without Xpert)	0.2	0.1–0.5	Model assumption
Public sector (without Xpert)	0.2	0.1–0.5	Model assumption
Xpert	0.73	0.6–0.8	[22]
**Probability of treatment success**			[1],[34]
First-line regimen/retreatment (not MDR-TB)	0.95	0.9–1.0	
First-line regimen/retreatment (MDR-TB)	0.53	0.4–0.6	
Second-line regimen (MDR-TB)	0.70	0.6–0.8	
**Proportion of MDR-TB cases successfully diagnosed as MDR-TB within 2 wk and put on second-line therapy**			[22]
Without Xpert	0	Unchanged	
With Xpert	0.94	0.8–1	
**Delay between diagnosis and treatment initiation**	0.5 mo	0–1 mo	Model assumption
**Self-cure rate, highly infectious TB (HIV-negative individuals only)**	0.1	0.01–0.1^c^	[47]
**Self-cure rate, less infectious TB (HIV-negative individuals only)**	0.27	0.1–0.3^c^	[47]

a Sensitivity analysis conducted by varying the transmission rate such that annual incidence changed by ±25%. In the multiway analysis, parameter combinations that resulted in more than a 25% change in baseline incidence were discarded.

b “Highly infectious” means “diagnosable by smear.” Individuals with highly infectious TB are assumed to be less infectious until seeking diagnosis. “Less infectious” means “not diagnosable by smear.”

c In multiway sensitivity analyses, some parameter values were made to correlate with each other so they either both increase or both decrease from their base value: (1) the proportion of infections that are highly infectious in those HIV− and those HIV+; (2) the proportion of individuals that progress rapidly in those HIV− and HIV+; (3) losses to follow-up between culture, Xpert, and smear; (4) endogenous activation of TB for those HIV− and HIV+; (5) the self-cure rate for highly infectious and less infectious TB.

d Transmission rate varied so that incidence remained constant.

e Where current diagnostic attempt is made in the informal sector, sensitivity range for next visit being to the qualified private sector is 0.25–0.75. The movement to the public sector is unchanged at 0.03, and remaining in the informal sector is the balancing figure (022–0.72). Where current diagnostic attempt is made in the qualified private sector, sensitivity range for next visit being to the public sector is 0.4–0.8. The movement to the informal sector is unchanged at 0.03, and remaining in the private sector is the balancing figure (0.17–0.57).

f Individuals with a history of TB treatment had an increased probability of diagnosis of half the difference between one and the probability of diagnosis for first-time infections (model assumption).

### MDR-TB, TB Treatment, and HIV

Since Xpert can detect resistance to rifampin [21],[30], we modeled infection with drug-susceptible and rifampin-resistant TB (as a proxy for MDR-TB) as separate strains, with rifampin resistance propagating through both inadequate treatment (potentially different across sectors) and transmission of resistant strains. In the absence of Xpert, we assumed that all newly diagnosed individuals receive first-line treatment lasting 6 mo, with an equal probability of success (reflecting notified success rates) across the public and qualified private sectors. In sensitivity analysis, we considered lower treatment success in the private sector [3],[31]–[33]. All cases with a history of TB treatment are initially placed on an 8-mo retreatment regimen. We assumed that half of individuals with MDR-TB fail these therapies and remain infectious [34]. A small proportion of cases with MDR-TB treatment failure are started on appropriate second-line therapy (20 mo). HIV infection accounts for a small minority (4.2%) of individuals with active TB in India [1]. Nevertheless, we included people living with HIV as a parallel population with higher TB risk and mortality.

### Model Calibration

We initiated our model at steady state, reflecting trends in India prior to 2005. Taking all other parameters as fixed, we fitted the following model parameters (Table S2): the transmission rate (number of transmitted infections per person-year), calibrated to the WHO-estimated overall TB incidence [1]; the duration of infectiousness before symptom onset, calibrated to the WHO-estimated duration of TB disease (prevalence/incidence ratio) [1]; and the probability of TB diagnosis in the qualified private sector, calibrated to estimates of the case notification proportion, assuming that non-notified cases are diagnosed and treated in the private sector [8].

We fit the model to the data shown in Table 3 in a model run to steady state using a quasi-Newton method [35]. After achieving steady state, we instituted a 2%/y decline in per capita TB incidence, to reflect the pre-Xpert (2005–2011) epidemiological situation in India [1].

**Table 3:** Model calibration.

Data point	Reported Value	Adjusted Value	Fitted Value	Source
Prevalence (per 100,000)	249	293	293	[1]
Annual incidence (per 100,000)	181	213	213	[1]
TB mortality (per 100,000)	24	29	29	[1]
Proportion of TB infections in HIV+ individuals	0.042	0.042	0.042	[1]
Proportion MDR-TB in all infections	0.021	0.021	0.021	[1]
Proportion of diagnoses made in qualified private sector	0.4	0.4	0.4	[8]

The reported values represent the estimated burden of TB in India. The adjusted values reflect the adult-only rates for pulmonary TB (the reported values represent all individuals and include pulmonary and extrapulmonary TB, whereas our model is an adult-only model of pulmonary TB). The fitted value represents the value we obtained in our model following our calibration exercise. Adjusted values were calculated using the fact that individuals aged 15 y and under represent 2% of notified cases and 30% of the population and that 85% of TB cases are pulmonary TB [1].

### Impact of Xpert

Where available, we assumed Xpert improved diagnosis and treatment as follows (numbers in parentheses denote sensitivity analysis ranges): increased sensitivity for less infectious (smear-negative) TB from 0%–20% (depending on health-care sector) to 73% (60%–80%); increased sensitivity to highly infectious TB from 0%–98% to a uniform 98% (80%–100%); reduced probability of loss to follow-up before treatment initiation for smear-negative TB (25%) to that of smear-positive TB (15%, with sensitivity analysis for 5%–25%), assuming that Xpert is rolled out with similar turnaround time to smear [1],[2],[36]; and increased sensitivity for rifampin resistance from 0% to 94% (80%–100%)

After fitting the starting model as above, we modeled six different Xpert rollout scenarios, comparing them to a baseline scenario of no Xpert access (Table 4). Each rollout scenario was run for 5 y. For each scenario, we selected 20% as an a priori coverage level that might be feasible at the country level, yet could have measureable population-level impact. The baseline scenario assumed no improved diagnostic testing. Scenario 1 (“public sector, HIV/high MDR-TB risk only”) assumed that 40% of individuals with TB symptoms presenting to the public sector who were either HIV-positive or had a history of TB treatment would be tested with Xpert [1],[3]–[5],[21],[27]. Scenario 2 (“broad public sector”) assumed Xpert access for 40% of individuals with HIV/high MDR-TB risk as above, plus 20% of all other individuals with TB symptoms (presumed TB) seeking diagnosis in the public sector (e.g., in upgraded peripheral microscopy centers). Scenario 3 (“qualified private sector”) assumed Xpert access for 40% of individuals with HIV/high MDR-TB risk in the public sector, as in scenario 1, plus 20% of all symptomatic individuals seeking care from qualified private practitioners (e.g., through private lab networks). Scenario 4 (“public plus qualified private sectors”) assumed access for the populations in both scenarios 2 and 3. Scenario 5 (“broad cross-sector access”) was designed to show the potential effect of Xpert distributed across all sectors, assuming Xpert access for 20% of all diagnostic encounters, including incentives for informal providers to refer patients to either the public sector (e.g., via public–private mix) or qualified private providers. Scenario 6 (“increased referral”) modeled the independent effect of incentivizing referrals from the informal sector to the public sector, without the added sensitivity of Xpert. Here, we assumed that 20% of individuals with TB who sought diagnosis in the informal sector were subsequently referred to the public sector for their next diagnostic attempt (up from 3% in the base case), with diagnosis made in the public sector by sputum smear microscopy.

**Table 4:** Scenario overview.

Scenario	Public Sector (High Risk for MDR-TB)	Public Sector (Low Risk for MDR-TB)	Qualified Private Sector	Informal Sector
Baseline	Sputum smear microscopy, no Xpert	Sputum smear microscopy, no Xpert	Existing mix of tests in private sector, no Xpert	Existing mix of tests in private sector, no Xpert
1. Public sector, HIV/high MDR-TB risk only	Baseline + Xpert for 40%	Baseline	Baseline	Baseline
2. Broad public sector	Baseline + Xpert for 40%	Baseline + Xpert for 20%	Baseline	Baseline
3. Qualified private sector	Baseline + Xpert for 40%	Baseline	Baseline + Xpert for 20%	Baseline
4. Public plus qualified private sectors	Baseline + Xpert for 40%	Baseline + Xpert for 20%	Baseline + Xpert for 20%	Baseline
5. Broad cross-sector access	Baseline + Xpert for 40%	Baseline + Xpert for 20%	Baseline + Xpert for 20%	Baseline + Xpert for 20%
6. Increased referral	Baseline	Baseline	Baseline	Baseline

The table shows the diagnostic algorithm used for each scenario to diagnose TB among individuals with respiratory symptoms in whom a diagnosis of TB is being considered. We do not consider active screening in this model.

To calculate the minimum number of GeneXpert systems required to conduct testing, we assumed that each system contained four modules, ran at a capacity of 16 tests per day, and operated 300 d per year (i.e., 4,800 tests per year). To conservatively estimate the total number of Xpert tests run, we assumed that 10% of all Xpert tests were conducted on individuals with underlying TB (i.e., 90% of tests were run on people without TB) in scenarios 2–4 [17],[24], increasing to 20% in scenario 1 and declining to 5% in scenario 5. Using these estimates, we also constructed analyses in which 100 GeneXpert systems were rolled out at maximum capacity, but across different sectors. As of December 31, 2013, the equivalent of 135 four-module GeneXpert systems had been procured in India under concessionary pricing [37].

### Sensitivity Analysis

Some data suggest that TB drug prescriptions are suboptimal [3],[4],[7] and treatment outcomes in the private sector may be inferior compared with the public sector [3],[31]–[33]. Therefore, we conducted an alternative analysis where we assumed lower-quality treatment in the private sector [3],[4],[11],[21],[32],[38]. In this analysis, individuals diagnosed and treated by qualified private practitioners had a 5-fold increase in the probability of developing MDR-TB during treatment (4% per treatment, versus 0.8% in the public sector) and a reduced probability of cure (75% versus 95%).

The broad rollout of Xpert in the public sector (scenario 2) may lead to behavioral changes in both patients and private providers that lead to increased care-seeking in, or referral to, the public sector, especially for MDR-TB, which is expensive to treat in the private sector. We therefore conducted a sensitivity analysis where the rollout of Xpert was accompanied by a 50% increase in seeking care within the public sector after a private-sector encounter.

We also conducted one-way sensitivity analyses using the deterministic model described above. We varied each model parameter in turn to the upper and lower bounds of the ranges given in Table 2 to assess the impact on model outcomes.

### Uncertainty Analysis

In addition to the sensitivity analyses above, to obtain measures of uncertainty that also included the impact of the random nature of both the disease and health-care behavior processes, we built a stochastic version of the model. We used a Gillespie stochastic simulation algorithm to incorporate stochasticity for each transition in our model [39]. The stochastic model used a population of 10 million individuals, and we conducted 10,000 runs of the model for each scenario. In each run, we simultaneously varied all parameter values using Latin hypercube sampling (i.e., probabilistic sensitivity analysis). We used a beta distribution with an alpha (shape) value of four and boundaries as described in Table 2 for each parameter. This approach therefore incorporated both parameter uncertainty and underlying stochastic uncertainty. Where we expected sets of parameters to be closely correlated, we varied their values in the same direction and magnitude within any simulation. We linked parameter values for TB natural history elements (e.g., proportion of rapid progression) for people with and without HIV, losses to follow-up across all diagnostic tests, and spontaneous resolution rates regardless of TB smear status, using correlation coefficients of 1.0 for transparency. All 95% uncertainty ranges (URs) reported in the manuscript reflect the 2.5th and 97.5th quantiles of the final distribution of outcomes under this procedure. To explore the independent influence of each parameter on the impact of Xpert after adjusting for the effects of all other parameters in the model, we calculated partial rank correlation coefficients (PRCCs) for the correlation between each parameter's value and key model outcomes [7],[40].

### Software

All analyses were conducted in R version 2.12 [41].

## Results

### Model Fit

The baseline model reflected estimates of the TB epidemic in India, with a TB incidence of 213 per 100,000 individuals per year among adults, prevalence of 293 per 100,000 (i.e., mean duration of disease as estimated by prevalence/incidence ratio = 1.38 y), TB mortality of 29 per 100,000 individuals per year, and MDR-TB prevalence of 2.1% among all incident active TB (Table 3). These values corresponded to a TB infection rate of 12.0 infections per highly infectious person-year, a total pre-diagnostic period (including time during which symptoms may be unnoticeably mild) of 9.0 mo, and probability of diagnosis in the qualified private sector of 38% per diagnostic attempt. The mean time from onset of infectiousness to first visiting an informal provider was 8.9 mo, increasing to 11.5 mo to visit to a qualified private provider and 14.8 mo to visit the public sector (among those who ever presented to the public sector).

### Impact of Xpert Scale-Up Scenarios

**Figure 2:**
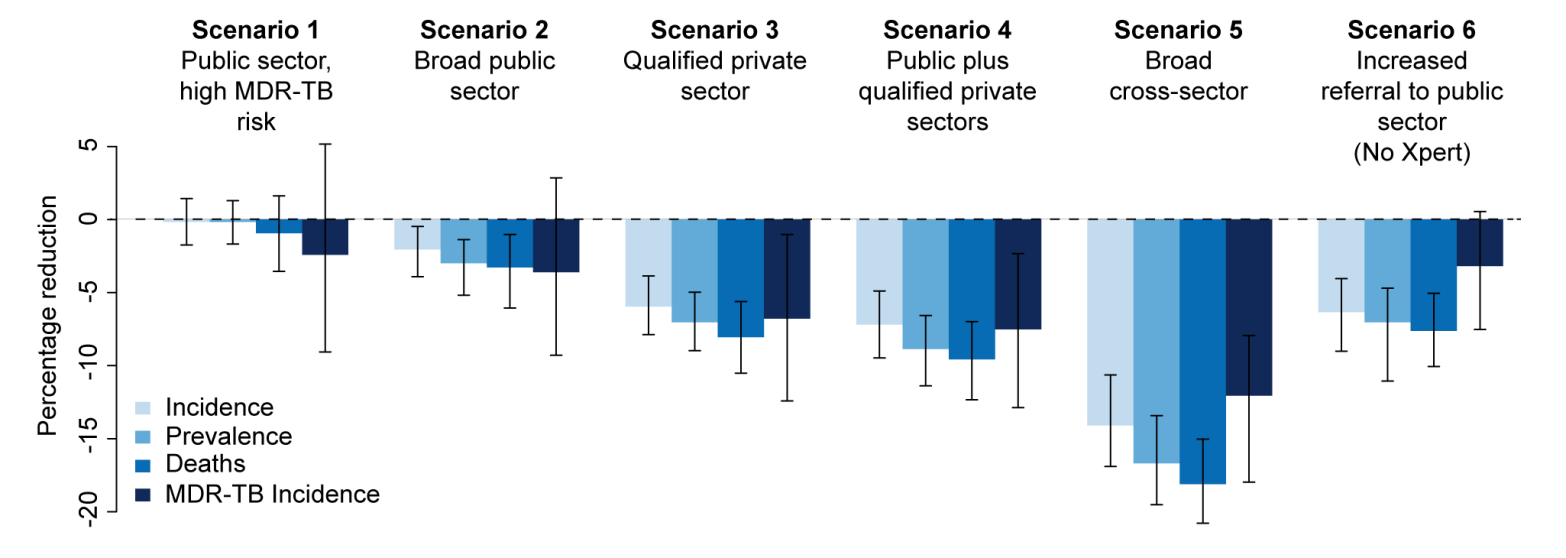
Impact of Xpert after 5 y. Percentage reduction in annual incidence, prevalence, mortality, and MDR-TB incidence from an Xpert rollout after 5 y in six different scenarios. The final set represents an alternative scenario where there is an increase in referrals from the informal sector to the public sector to 20% with no Xpert rollout.

In the baseline scenario (no new diagnostic intervention), TB incidence fell from 213 to 192 per 100,000 individuals per year over 5 y (i.e., continued 2% annual decline), whereas MDR-TB prevalence remained stable. Introducing Xpert into the public sector for 40% of HIV-positive individuals and individuals at high risk of MDR-TB (scenario 1) had a negligible epidemiological impact, with the uncertainty ranges for the effect on each of the epidemiological measures (incidence, prevalence, mortality, and MDR-TB incidence) including zero (Figure 2). We estimated that TB incidence in this scenario would fall slightly (192 per 100,000 individuals per year, a 0.2% decline relative to baseline, 95% UR: −1.4%, 1.7%); however, it would enable 14,000 additional true-positive MDR-TB diagnoses over a 5-y time period. If these individuals were appropriately treated, the impact on MDR-TB (from 4.4 to 4.2 per 100,000 individuals per year, a 2.4% decline, 95% UR: −5.2%, 9.1%) and TB mortality (0.9% decline, 95% UR: −1.6%, 3.5%) was more substantial (Table 5). Assuming that Xpert could be performed at near-maximum capacity (16 tests per machine-day, 300 d/y), such an implementation at the country level would require continuous use of 60 four-module GeneXpert systems in centralized laboratories—about half of the number of modules procured country-wide through 2013. Using Xpert on smear-positive specimens only would lower this requirement to 11 systems while preserving two-thirds of the impact on MDR-TB incidence (1.7% decline), but sacrificing most of the impact on TB mortality (0.02% reduction).

**Table 5:** Effect of Xpert rollout on annual TB incidence and mortality after 5

Scenario	Cases Averted^a^	Deaths Averted^a^	MDR-TB Cases Averted^a^^,^ b	Additional MDR-TB Diagnoses (×10^3^/y, All India^a^^,^ c)	Total Number of Xpert Tests Conducted (×10^3^/y, All India^a^^,^ c ^,^ d)	Minimum Number of Xpert systems Required (All India^a^^,^ c ^,^ e)
1. Public sector, HIV/high MDR-TB risk only	0.2% [−1.4, 1.7] (0.3 per 100,000)	0.9% [−1.6, 3.5] (0.2 per 100,000)	2.4% [−5.2, 9.1] (0.1 per 100,000)	2.5 [1.4, 4.4]	300 [250, 420]	60 [50, 90]
2. Broad public sector	2.1% [0.5, 3.9] (4.0 per 100,000)	3.3% [1.0, 6.1] (0.9 per 100,000)	3.6% [−2.9, 9.3] (0.2 per 100,000)	4.7 [3.0, 6.6]	3,200 [2,400, 4,000]	700 [490, 840]
3. Qualified private sector	6.0% [3.9, 7.9] (11.5 per 100,000)	8.1% [5.6, 10.5] (2.1 per 100,000)	6.8% [1.0, 12.4] (0.3 per 100,000)	5.9 [3.9, 7.7]	3,500 [2,600, 3,900]	700 [530, 810]
4. Public plus qualified private sectors	7.2% [4.9, 9.5] (13.9 per 100,000)	9.6% [7.0, 12.3] (2.5 per 100,000)	7.5% [5.7, 10.6] (0.3 per 100,000)	7.2 [5.0, 9.1]	5,100 [3,700, 5,800]	1,100 [770, 1,220]
5. Broad cross-sector access	14.1% [10.6, 16.9] (27.2 per 100,000)	18.1% [15.0, 20.8] (4.7 per 100,000)	12.1% [7.9, 18.0] (0.5 per 100,000)	7.7 [5.2, 9.2]	10,800 [8,000, 12,100]	2,200 [1,670, 2,510]
6. Increased referral	6.3% [4.0, 9.0] (12.2 per 100,000)	7.6% [5.0, 10.1] (2.0 per 100,000)	3.2% [−0.6, 7.5] (0.1 per 100,000)	—	—	—

a 95% uncertainty ranges provided in square brackets.

b Assuming second-line treatment is available for those diagnosed with MDR-TB.

c Estimated population of India by 2019 is 1.3 billion.

d Assumes 20% of Xpert tests are performed on individuals with TB for scenario 1, 10% for scenarios 2–4, and 5% for scenario 5.

e Assumes four runs per day per module and each machine has four modules and operates 300 d/y.

If Xpert was made available to 20% of symptomatic individuals seeking care in the public sector (in addition to 40% of individuals with HIV/high MDR-TB risk as above) (scenario 2), incidence fell to 189 per 100,000 individuals per year (2.1% decline relative to baseline, 95% UR: 0.5%, 3.9%) over 5 y, with correspondingly larger relative effects on TB mortality (25 per 100,000 individuals per year, 3.3% decline, 95% UR: 1.0%, 6.1%) and MDR-TB incidence (4.1 per 100,000 individuals per year, 3.6% decline, 95% UR: −2.9%, 9.3%). However, such an implementation would require 3.2 million annual tests, or 700 continuously running GeneXpert systems, approximately one per district (India has 680 districts), and over five times as many systems as had been procured via concessionary pricing through 2013.

Providing Xpert access to the qualified private sector (scenario 3) had greater impact on incidence (6.0% reduction, 95% UR: 3.9%, 7.9%), and with similar resource requirements (700 systems), assuming treatment success equivalent to that in the public sector. Correspondingly, broad access to Xpert in both public and private sectors (scenario 4) produced only a slight incremental impact compared to access in the qualified private sector only, with overlapping uncertainty ranges between the two scenarios in each of the epidemiological measures (Figure 2), reflecting the fact that nearly all individuals diagnosed in the public sector were previously seen by qualified private providers.

**Figure 3:**
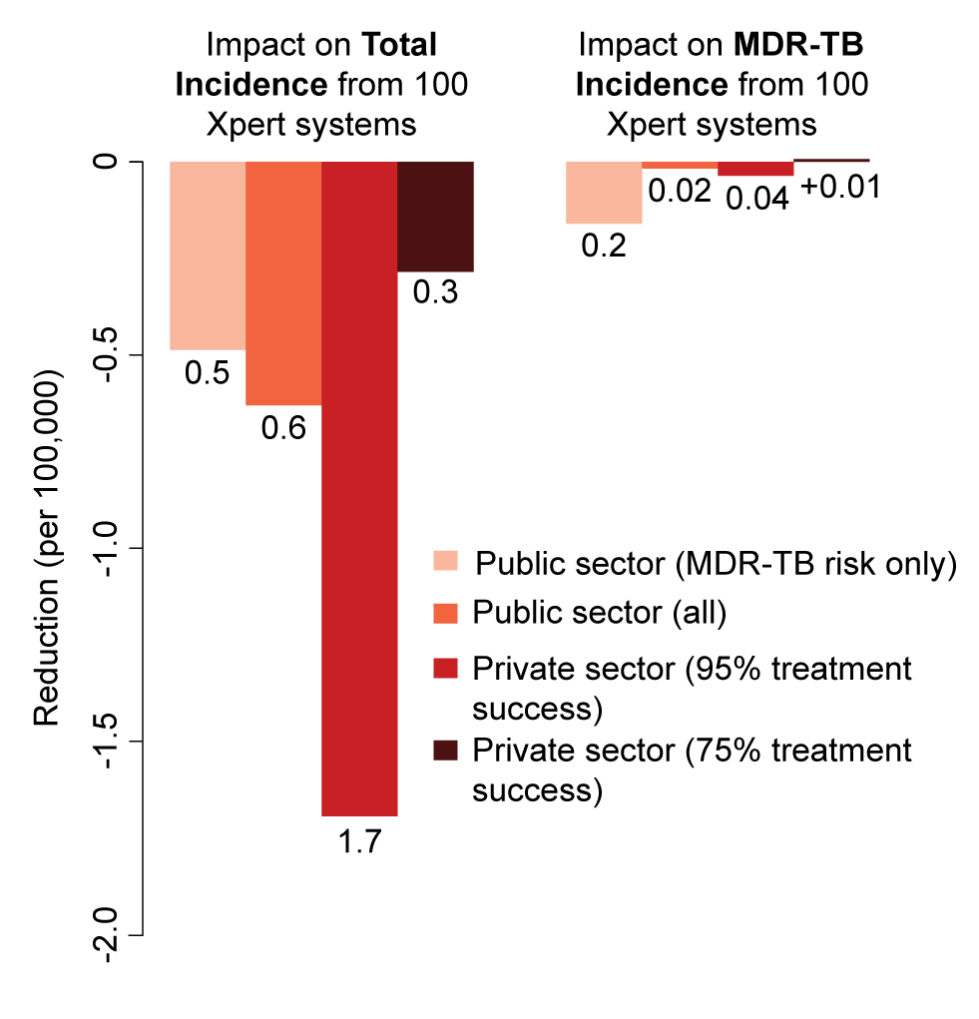
Impact of 100 Xpert systems rolled out in different sectors after 5 y. Reduction in total annual incidence and MDR-TB incidence per 100,000 individuals from a rollout of 100 Xpert machines. The scenarios are as described in the Methods. Rollout of 100 Xpert machines in the private sector has substantially greater impact than a similar rollout in the public sector, but only if high treatment success can be assured. If treatment is poor, use of Xpert machines in the private sector has no epidemiological benefit.

**Figure 4:**
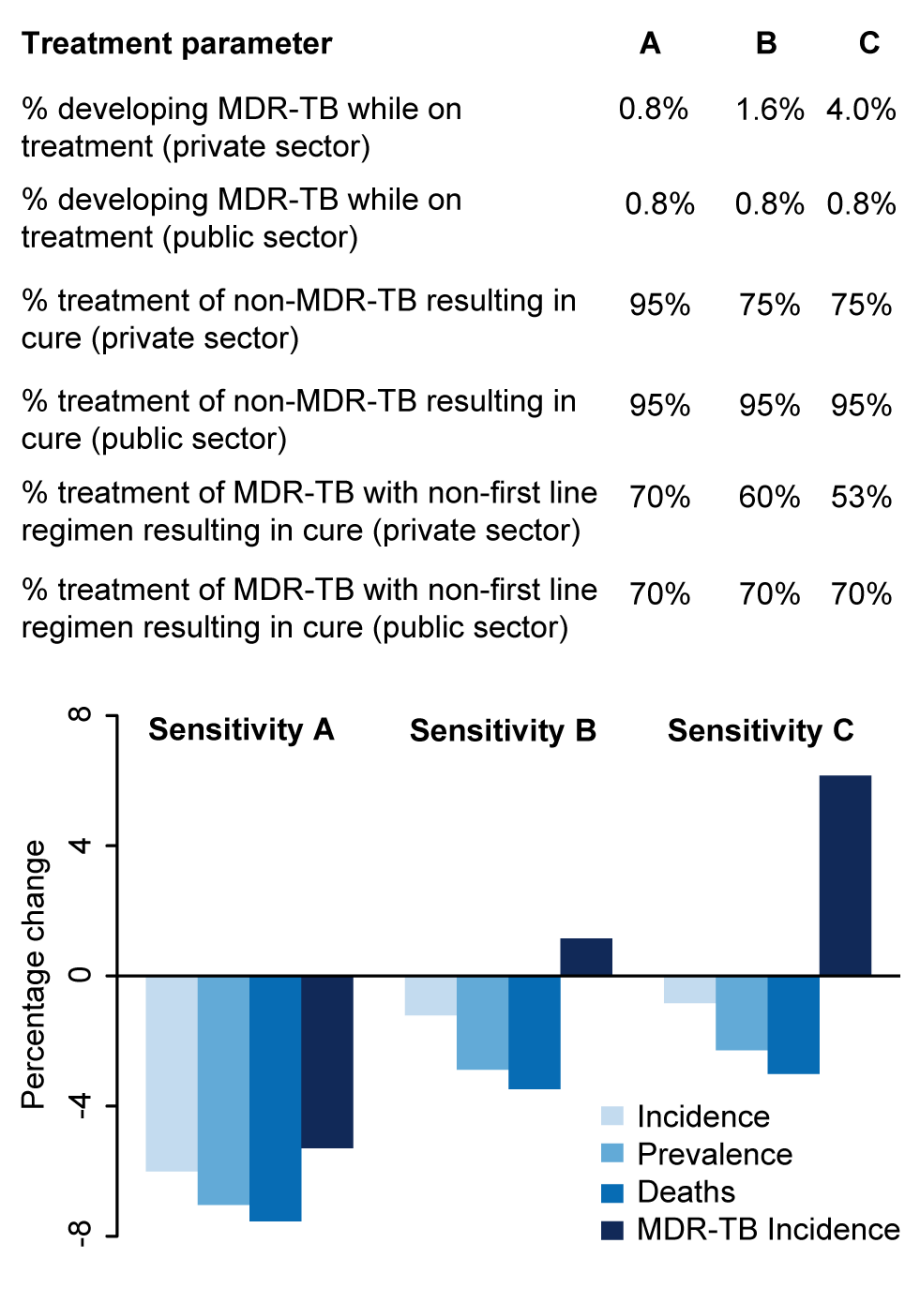
Impact of differential treatment failure between public and private providers. The qualified private sector represents a wide range of operations, many of which are believed to provide poor levels of treatment. To explore the potential impact of a rollout of Xpert access for 20% of patients seeking care in the qualified private sector where the treatment provided in the qualified private sector is poorer than that provided in the public sector, we ran three sensitivity analyses. Sensitivity A represents the main analysis with no difference between the private and public sectors. In Sensitivity B, patients put on treatment in the private sector have twice the probability of developing MDR-TB as those put on treatment in the public sector, and lower levels of treatment success. In Sensitivity C, patients put on treatment in the private sector have five times the probability of developing MDR-TB as those put on treatment in the public sector, and lower levels of treatment success.

In analyses of rolling out 100 maximum-capacity GeneXpert systems across different sectors, Xpert access in the qualified private sector had more than twice the impact on overall incidence of access in the public sector, whereas targeting high-risk individuals had 5–10 times more impact on MDR-TB than non-targeted strategies (Figure 3). Assuming poorer treatment outcomes in the private sector caused the relative benefit of private-sector Xpert access to disappear (Figure 4).

Where we considered incorporating access to all providers—assuming referral from the informal sector to other providers for appropriate treatment (scenario 5)—such broad-based access dramatically increased the impact on incidence (14.1% reduction, 95% UR: 10.6%, 16.9%), but at substantial additional resource requirement (2,200 Xpert systems required). As an alternative to Xpert rollout, simply increasing access to public-sector smear diagnosis (scenario 6) had a large effect: if 20% of symptomatic individuals seeking care at informal providers were simply referred to the public sector on their next attempt, incidence was reduced by 6.3% without any use of Xpert at all (Figure 2).

### Sensitivity Analyses

**Figure 5:**
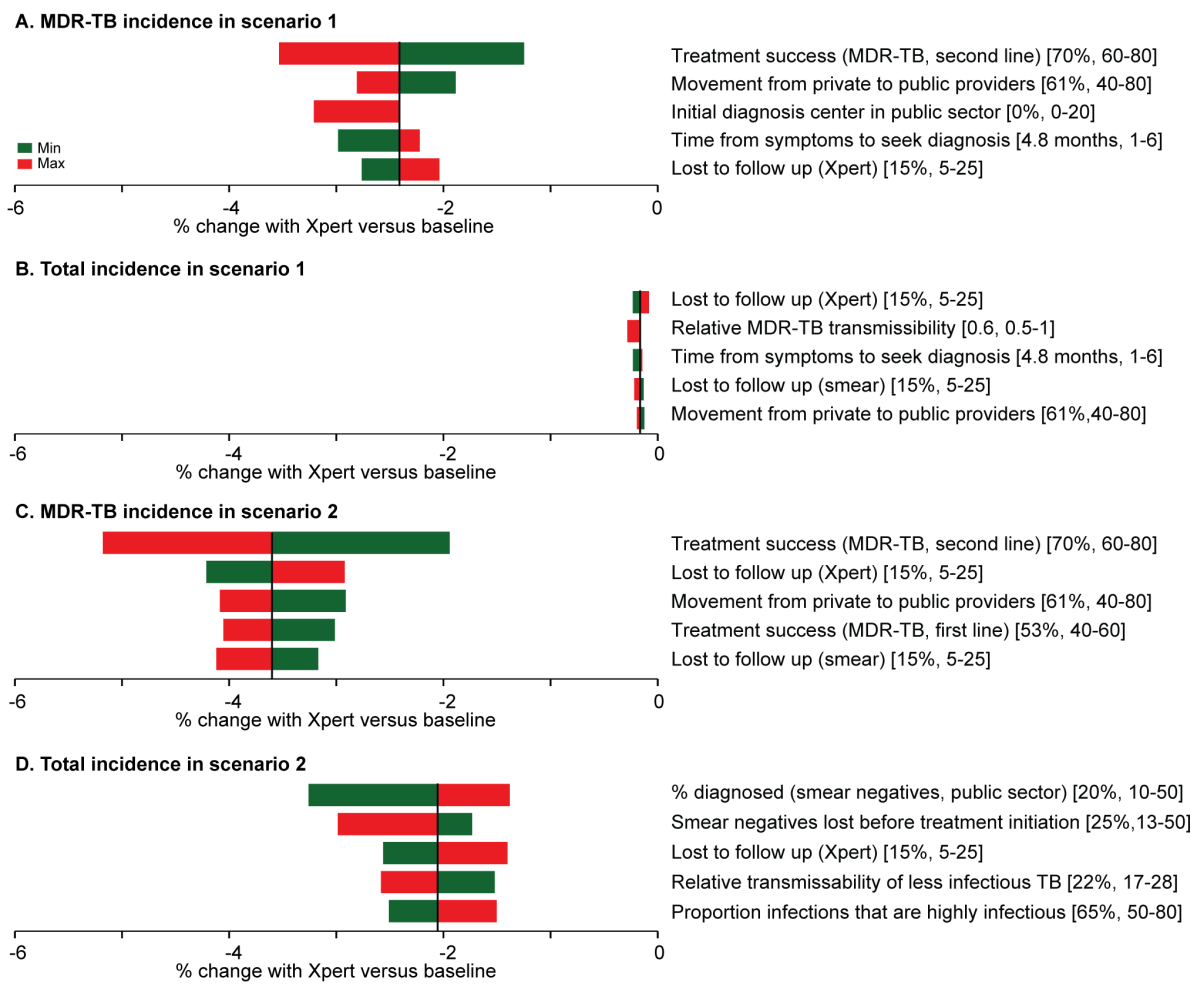
One-way analysis of parameter sensitivity. The parameters were changed in turn to the maximum (red) and minimum (green) values from Table 2. The effect of Xpert after 5 y on MDR-TB incidence and overall incidence in scenarios 1 ([A] and [B]) and 2 ([C] and [D]) was recorded for each new value. The five parameters to which the model is most sensitive are shown in the diagrams. In both cases, the most important parameters in one-way sensitivity analysis reflected aspects of the existing health-care system in India, not characteristics of the diagnostic assay itself.

**Figure 6:**
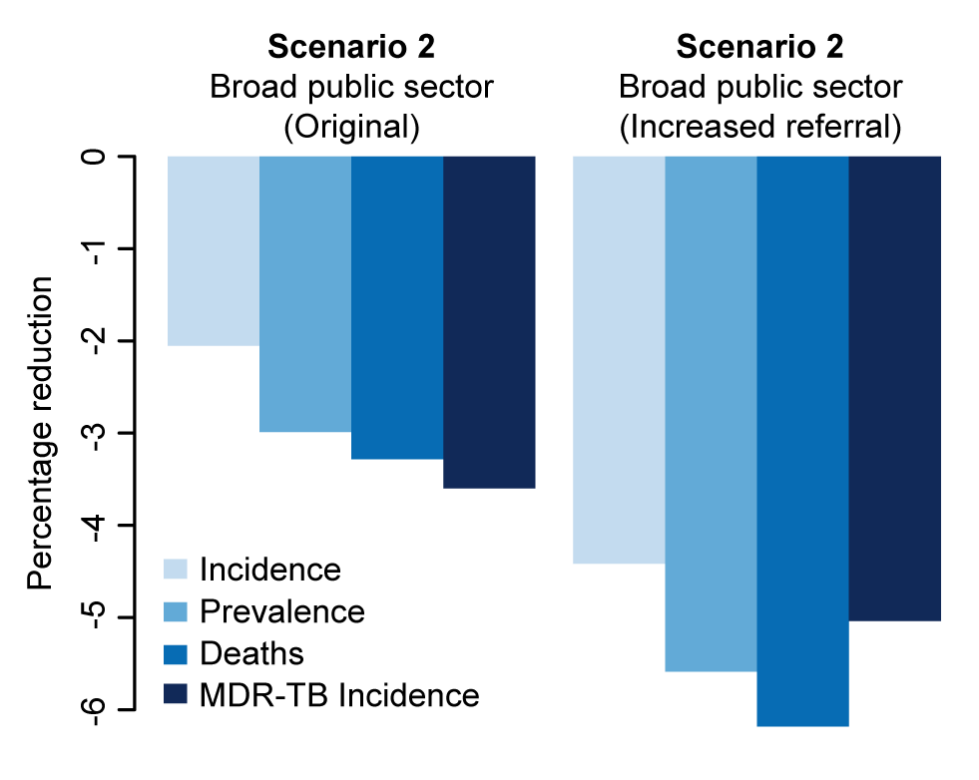
Impact of behavioral changes. We explored the impact of increasing referrals from the informal and qualified private sectors to the public sector following broad access to Xpert in that sector (scenario 2). The figure shows the impact on incidence of a 50% increase in the referral rate from qualified private and informal providers to the public sector where Xpert is broadly available in the public sector but not available in the private sector (scenario 2).

In both one-way analyses (Figure 5) and sensitivity analyses that adjusted for the simultaneous effects of all other parameters (Figure S2), the effect of Xpert for people with HIV or previous TB treatment (scenario 1) on MDR-TB incidence was most sensitive to the success of second-line therapy for patients with MDR-TB. If second-line therapy cured only 60% of MDR-TB cases, Xpert would reduce MDR-TB incidence by only 1.2% after 5 y (Figure 5A). The sensitivity analyses also agreed that the impact of Xpert in the public sector (scenario 2) was most sensitive to the baseline probability of diagnosis in the public sector: increasing the diagnosis probability for less infectious (smear-negative) TB from 10% to 50% changed the impact of Xpert on TB incidence from 3.3% to 1.4% (Figure 5D). The transmission fitness of MDR-TB did not substantively affect the relative impact of Xpert on MDR-TB incidence (PRCC −0.01), nor did wide variation in the structure of care-seeking attempts after the initial visit (e.g., 25%–75% probability of seeking care in the qualified sector after an informal sector attempt, PRCC 0.35) (Figure S2). Including an additional active disease compartment resulted in identical estimates of the impact of Xpert across all scenarios, reflecting the near-equilibrium conditions of the baseline scenario (Text S3). If broad public-sector rollout increased the probability of seeking care in the public sector (after a private-sector encounter) by 50%, incidence fell by an estimated 4.4%, greater than the 2.1% estimated with no behavior change, but still lower than the impact of rollout to the qualified private sector (Figure 6).

## Discussion

Xpert is more sensitive than other diagnostics in current widespread use, but its cost is substantial, and the Indian health-care system is fragmented and heavily privatized. These circumstances pose challenges for the design of an optimal implementation strategy. Currently, the RNTCP is mainly implementing Xpert as a rapid DST method among high-risk cases seeking care in the public sector; our model suggests that the impact of this strategy on the overall TB epidemic in India will be limited. By contrast, scale-up of Xpert access to include qualified providers (e.g., through private lab networks), or improving referrals from informal to public providers with no Xpert at all, could have a substantial impact (6% reduction in incidence after 5 y, or over 30,000 lives saved per year, three times more than a public-sector Xpert rollout of similar scope). In addition, the only rollout scenarios that produced reductions in MDR-TB incidence in which the 95% uncertainty range excluded zero were those that provided Xpert access to the private sector (and assumed high-quality treatment within that sector).

The impact of Xpert for TB control in India therefore depends not only on test accuracy, but also on the implementation strategy, reliable linkages to high-quality treatment, and commitment of resources. These findings are relevant to policy in India, and more broadly in implementing the National Strategic Plan (2012–2017) [16] for TB made by the RNTCP that considers the most appropriate way to scale up Xpert or better alternatives that may emerge in the future.

A key consideration of any Xpert implementation strategy will be the costs associated with each approach. Costs will vary depending on a large number of factors including machine placement (decentralized versus centralized network of laboratories), transportation and maintenance costs, future pricing schemes, development of next-generation diagnostic tests, costs of providing free MDR-TB treatment in the public sector, and costs to patients (both direct and indirect). Thus, we focus here on the number of GeneXpert systems required to achieve various rollout strategies, as a proxy for required resources. In generating these estimates, we assumed that each module would perform four tests a day, but this number may be optimistic, as module failure, cartridge stock-outs, laboratory logistics (e.g., specimen transport), and insufficient numbers of samples or staff could all reduce the daily capacity of an Xpert module. A recent pilot study in India processed two samples per module per day [25]; if this capacity is applied to all implemented modules, our estimates of the number of four-module GeneXpert systems required double. Even using our optimistic assumptions, however, the number of systems required even for our broad public-sector or qualified private-sector scenarios (700 four-module systems, or over US$10 million in equipment prices alone, with cartridges costing over three times more) are unlikely to be realized in the short term without dramatic increases in funding. This funding, however, need not come entirely from the public sector; given the potential for Xpert to have even greater epidemiological impact if deployed in the private sector, innovative mechanisms should be pursued to replace existing resource outlays in the private sector (e.g., using Xpert rather than non-approved tests for active TB) and to speed patient access (e.g., using referral systems or specimen transport networks) to high-quality TB diagnosis throughout India, without saddling patients with extra out-of-pocket costs. Such systems are likely to be developed only with strong political will and effective engagement of a diverse spectrum of stakeholders in the Indian health-care system.

To provide perspective on resource requirements, through 2013, the country had acquired the equivalent of 135 four-module GeneXpert systems via concessionary pricing [37], less than one-fifth that required for our 20% scale-up scenarios. Treatment of the 43,800 additional patients found to have MDR-TB over 5 y would require even greater resource outlay. Thus, for Xpert to have substantial population-level impact in India, the private sector must be engaged, but a dramatic increase in resource allocation is also essential. These resources need not come only from the public sector; engaging private lab networks may make Xpert more affordable in the private sector as well [1],[4],[8],[10]–[12],[20],[42]. One model for such engagement is the Initiative for Promoting Affordable and Quality TB Tests (http://www.ipaqt.org/), through which nearly 50 Xpert systems have been installed in private labs across India that offer WHO-endorsed tests at more affordable prices in the private sector [20],[42]. Furthermore, to the extent that private-sector resources can be diverted from inappropriate diagnostic tests (e.g., serology and IGRAs for active TB) to high-quality tests of similar price (e.g., Xpert), implementation of Xpert in India has the potential to be both less costly and more effective than the current standard of care in TB diagnosis.

A previous model of Xpert rollout in southern Africa estimated a 6% reduction in annual incidence after 10 y [26], similar to our scenario 3, with 20% access in the private sector. Our “idealized access” scenario (scenario 5) projects greater impact on incidence (14% reduction after 5 y), reflecting lower levels of HIV co-infection, but would be so resource-intensive as to be currently unrealistic. Another model of hypothetical TB diagnostics in a generic Southeast Asian context [43] found that sensitivity and point-of-care amenability—as characteristics of such hypothetical assays—represented tradeoffs in terms of impact; by contrast, the present model is fit specifically to the Indian epidemiological and health-care system, evaluates realistic scale-up scenarios of an actual diagnostic test (Xpert) being actively discussed at the policy level in India, and includes country-specific data on patient pathways to care. We show that these aspects of the health-care system, patient and provider behavior, and implementation strategy are at least as important—if not more so—than specific assay characteristics when evaluating the potential impact of TB diagnostic tests in India.

This model represents a first attempt to explore the interactions between rollout of a novel TB diagnostic test and a complex underlying health-care system, highlighting how patient and provider practices may affect TB incidence in the population. For Xpert or any other diagnostic test to reach maximum potential in India, engaging the private sector and assuring high-quality care will be critical; scenarios that included only the public sector or assumed poor treatment success proved inadequate for TB control. Facilitating referrals from the informal sector to the public sector (e.g., through innovative incentive schemes, public–private interface agencies [PPIAs], or social franchising) [44] can also have a dramatic impact, rivaling that of any Xpert rollout. The RNTCP's National Strategic Plan for TB in India includes a PPIA component to improve quality of care in the private sector and increase referrals to the public sector [16]; two urban pilot PPIA projects are underway [44]. Although India's TB diagnostic pathways are in many ways unique, our quantitative estimates reflect that unique system, and our key findings (e.g., importance and resource intensity of engaging the private sector) may generalize to other countries—especially those in Southeast Asia and the Western Pacific—with large private sectors and persistently high TB incidence despite relatively low levels of HIV and drug-resistant TB on a population level.

As with any mathematical representation, our model has certain limitations. It does not account for difficulties in implementation, inaccessibility or high costs of high-quality MDR-TB treatment, and nuanced HIV dynamics. Model results also reflect the quality of underlying data, and there remain key uncertainties regarding symptoms and infectiousness among TB patients. In addition, TB prevalence among people seeking care (with and without Xpert availability) remains poorly understood. Individuals with more severe symptoms may present at the public sector earlier, which might increase the impact of public-sector Xpert rollout on mortality. We conducted multiple sensitivity analyses on all parameters and found our results to be largely robust to parameter misspecification, although our uncertainty ranges are wide, reflecting uncertainty in the underlying data.

Understanding care-seeking pathways is essential to understanding the impact of novel diagnostics in the real world. As data on care-seeking behavior in the private sector are sparse, our estimates are based on a single cross-sectional study of patients who ultimately presented to the public sector. While we varied these estimates using wide uncertainty ranges, this work highlights important gaps in our knowledge, including the need for better data on the amount of time spent before seeking diagnosis, patient care-seeking pathways upon seeking diagnosis, and the quality of TB care in the private sector.

In conclusion, Xpert (and by extension, other novel diagnostics with similar or improved characteristics) could substantially reduce the burden of TB disease due to poor diagnosis in India; however, this impact depends not only on the accuracy of the test, but also on the behavior of both patients and providers, their level of access to new tools, and quality TB treatment following diagnosis. As such, any Xpert rollout strategy must also consider the complex health-care infrastructure into which the test is being rolled out. To achieve maximum impact of novel diagnostics, India should engage the private sector, improve quality of care across all sectors, and dramatically increase resources.

## Supporting Information

Figure S1 **Detailed model schematic.**
(PDF)

Figure S2 **Partial rank correlation coefficients.** To explore the independent influence of each parameter on the impact of Xpert we calculated the PRCC from 10,000 simulations, where all parameters were varied over the range set out in Table 2. PRCCs are adjusted for the simultaneous effects of all other parameters in the model. (A) sets out the ten parameters that had the highest PRCCs on MDR-TB incidence in scenario 1. (B) sets out the ten parameters that had the highest PRCCs on total incidence in scenario 2. Simulations that resulted in a greater than 25% change in total incidence were discarded and not included in the analysis.
(PDF)

Table S1 **Model compartments.** The model is composed of eight compartment types, subdivided by HIV status, MDR-TB status, smear status, infection parity, health-care provider type, whether treatment is successful, and treatment regimen.
(PDF)

Table S2 **Model parameters.** Overview of all model parameters.
(PDF)

Text S1 **Details of derived parameters.**
(PDF)

Text S2 **Model equations with supporting text.**
(PDF)

Text S3 **Sensitivity using gamma-distributed waiting time.**
(PDF)

## Abbreviations

DST: drug susceptibility testing
IGRA: interferon-gamma release assay
MDR-TB: multidrug-resistant tuberculosis
PPIA: public–private interface agency
PRCC: partial rank correlation coefficient
RNTCP: Revised National Tuberculosis Control Programme
TB: tuberculosis
UR: uncertainty range
Xpert: Xpert MTB/RIF
