# Supplementary material for: The Importance of Implementation Strategy in Scaling Up Xpert MTB/RIF for Diagnosis of Tuberculosis in the Indian Health-Care System: A Transmission Model
Source: PLoS Med. 2014 Jul 15;11(7):e1001674. doi: 10.1371/journal.pmed.1001674 (PMC4098913; doi:10.1371/journal.pmed.1001674)
Supplement: Figure S1 — Detailed model schematic. (PDF) [file pmed.1001674.s001.pdf]

# Figure S1. Model Schematic

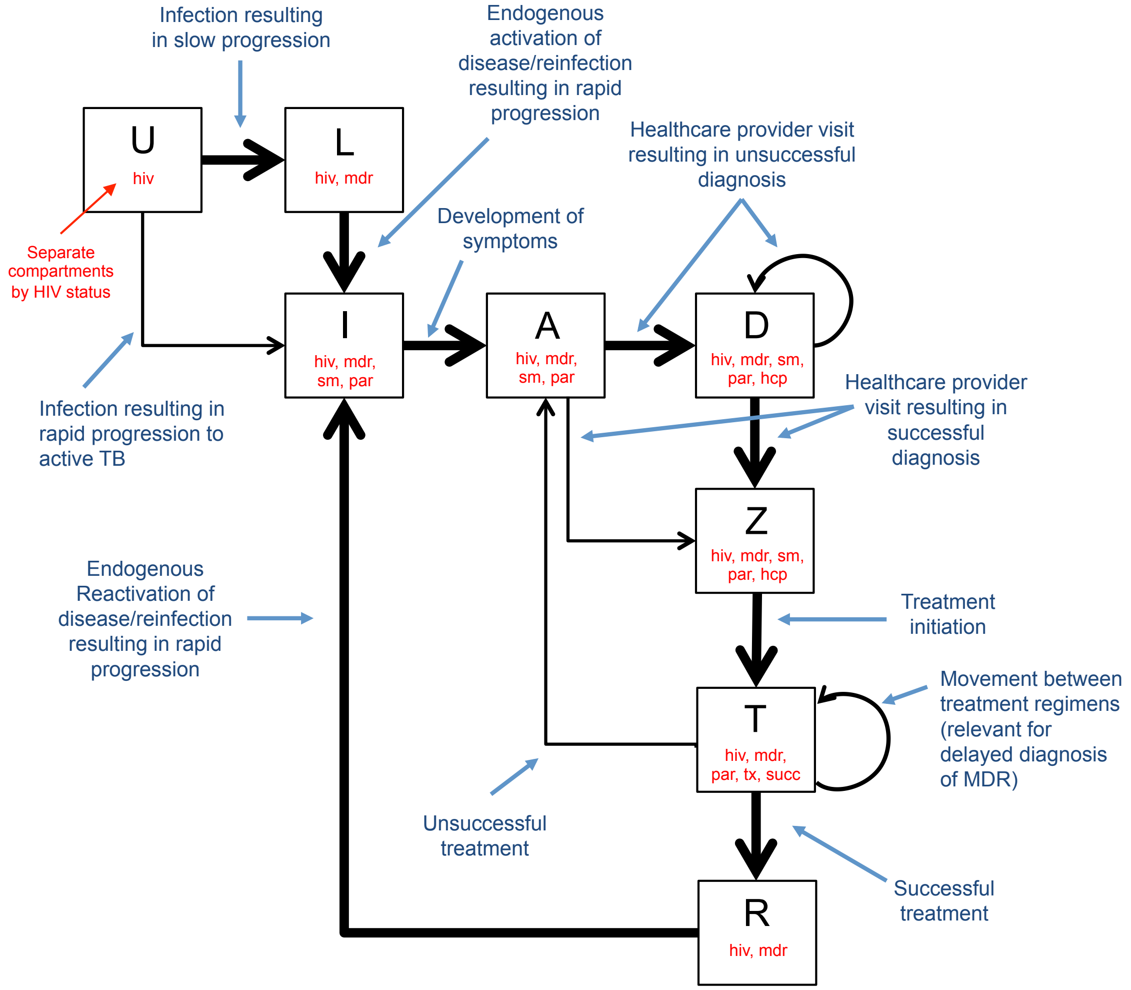


*sm* is smear status (positive or negative), *par* is treatment parity (either never treated or history of treatment), *hcp* is healthcare provider (informal, private qualified or public), *tx* is treatment regimen and *succ* is success of treatment.
