# Supplementary material for: The Importance of Implementation Strategy in Scaling Up Xpert MTB/RIF for Diagnosis of Tuberculosis in the Indian Health-Care System: A Transmission Model
Source: PLoS Med. 2014 Jul 15;11(7):e1001674. doi: 10.1371/journal.pmed.1001674 (PMC4098913; doi:10.1371/journal.pmed.1001674)

Figure S2. Partial rank correlation coefficients

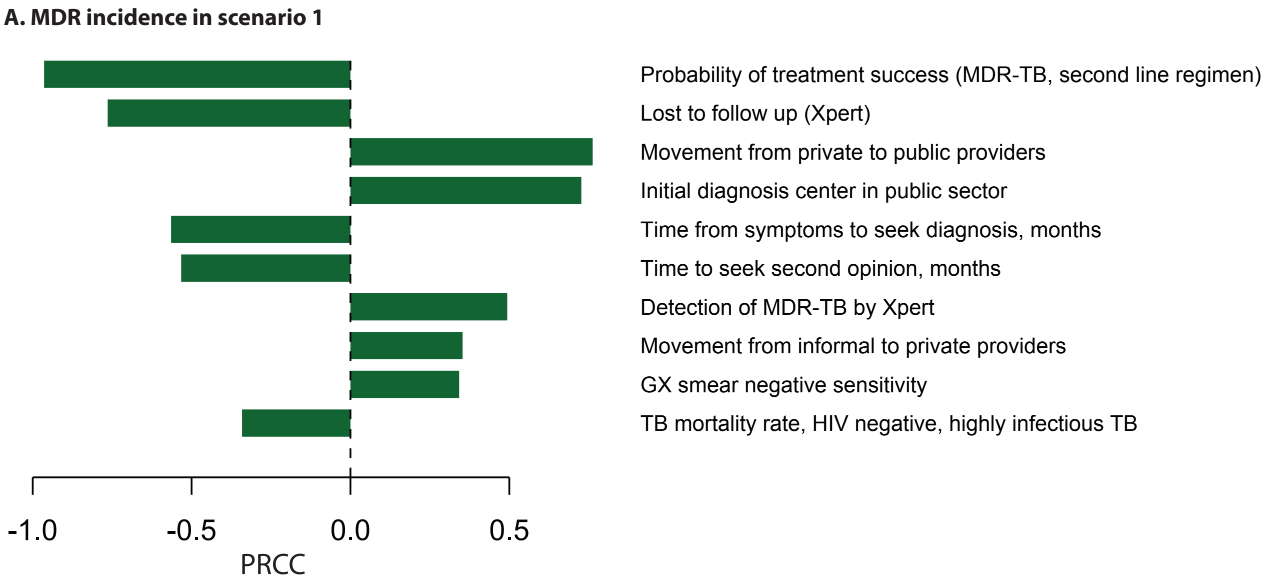

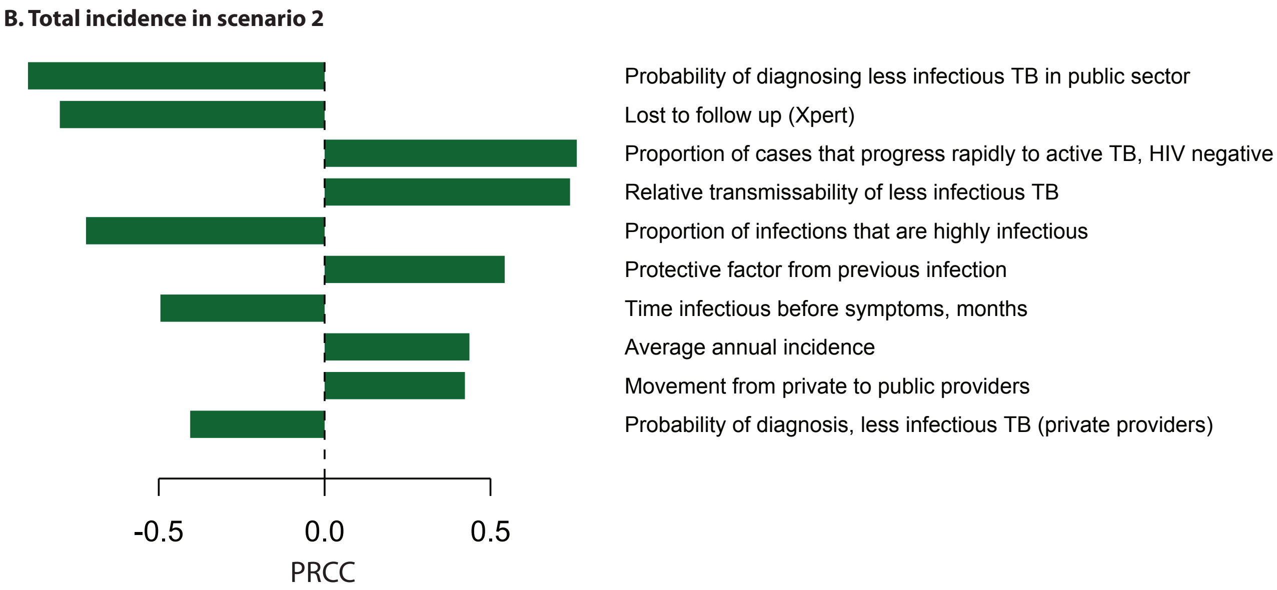

Supplement: Figure S2 — Partial rank correlation coefficients. To explore the independent influence of each parameter on the impact of Xpert we calculated the PRCC from 10,000 simulations, where all parameters were varied over the range set out in Table 2. PRCCs are adjusted for the simultaneous effects of all other parameters in the model. (A) sets out the ten parameters that had the highest PRCCs on MDR-TB incidence in scenario 1. (B) sets out the ten parameters that had the highest PRCCs on total incidence in scenario 2. Simulations that resulted in a greater than 25% change in total incidence were discarded and not included in the analysis. (PDF) [file pmed.1001674.s002.pdf]
