# Supplementary material for: The Importance of Implementation Strategy in Scaling Up Xpert MTB/RIF for Diagnosis of Tuberculosis in the Indian Health-Care System: A Transmission Model
Source: PLoS Med. 2014 Jul 15;11(7):e1001674. doi: 10.1371/journal.pmed.1001674 (PMC4098913; doi:10.1371/journal.pmed.1001674)
Supplement: Table S1 — Model compartments. The model is composed of eight compartment types, subdivided by HIV status, MDR-TB status, smear status, infection parity, health-care provider type, whether treatment is successful, and treatment regimen. (PDF) [file pmed.1001674.s003.pdf]

Table S1. Model compartments

Compartment	Description	Separated by
**U**	**Uninfected individuals**. Those never infected.	-HIV(+/-)
**L**	**Latently infected**. Infected individuals that progress slowly to active disease.	-HIV(+/-)<br>-MDR(+/-)
**I**	**Pre-symptomatic Infectious**. Infectious individuals that do not yet have symptoms.	-HIV(+/-)<br>-MDR(+/-)<br>-Smear(+/-)<br>-Treatment History (Never treated, Treated previously)
**A**	**Symptomatic active disease**. Symptomatic individuals with active disease who have not yet started seeking healthcare.	-HIV(+/-)<br>-MDR(+/-)<br>-Smear(+/-)<br>-Treatment History (Never treated, Treated previously)
**D**	**Diagnosis seeking**. Those that have visited healthcare providers for diagnosis but have not been successfully diagnosed.	-HIV(+/-)<br>-MDR(+/-)<br>-Smear(+/-)<br>-Treatment History (Never treated, Treated previously)<br>-HCP(informal/qualified/public)
**Z**	**Successfully diagnosed**. Those that have visited healthcare providers for diagnosis and have been successfully diagnosed and are awaiting treatment initiation.	-HIV(+/-)<br>-MDR(+/-)<br>-Smear(+/-)<br>-Treatment History (Never treated, Treated previously)<br>-HCP(informal/qualified/public)
**T**	**On treatment**. Those that are on treatment - either on first line (first time on treatment), retreatment (subsequent infections) or second line (after successful MDR diagnosis).	-HIV(+/-)<br>-MDR(+/-)<br>-Treatment History (Never treated, Treated previously)<br>-Treatment Regimen(first line/retreat/ second line)<br>-Success (cured/not cured)
**R**	**Recovered**. Those that have been cured by treatment or spontaneously recover. Susceptible to endogenous reactivation or new infection.	-HIV(+/-)<br>-MDR(+/-)

HCP: Healthcare Provider
