# Supplementary material for: The Importance of Implementation Strategy in Scaling Up Xpert MTB/RIF for Diagnosis of Tuberculosis in the Indian Health-Care System: A Transmission Model
Source: PLoS Med. 2014 Jul 15;11(7):e1001674. doi: 10.1371/journal.pmed.1001674 (PMC4098913; doi:10.1371/journal.pmed.1001674)
Supplement: Table S2 — Model parameters. Overview of all model parameters. (PDF) [file pmed.1001674.s004.pdf]

Table S2. Model parameters

Note on subscripts

Compartments and parameter values may be divided by one or more of HIV status, MDR status, smear status, infection parity, treatment regimen and healthcare provider. In each case, the subscript determines the type:

HIV positive:	$hiv = 1$	First line treatment:	$tx = fl$
HIV negative:	$hiv = 0$	Retreatment regimen:	$tx = rt$
MDR TB:	$mdr = 1$	Second line regimen:	$tx = sl$
non MDR-TB:	$mdr = 0$	Informal provider:	$hcp = inf$
Smear positive TB:	$sm = 1$	Qualified private provider:	$hcp = prv$
Smear negative TB:	$sm = 0$	Public provider:	$hcp = pub$
Never treated:	$par = 0$	Treatment resulting in cure:	$succ = 1$
Treatment history:	$par = 1$	Treatment not resulting in cure:	$succ = 0$

**Parameter**	**Symbol**	**Baseline**	**Source**
Transmission rate of smear positive TB (transmissions per year)	β	11.86 (solved)	
Baseline force of infection, by year	$\lambda_{mdr=0}$	Derived (see detail)	
Force of infection from MDR TB, by year	$\lambda_{mdr=1}$	Derived (see detail)	
Reduced transmissibility of smear negative TB	κ	0.22	[54]
Transmissibility of MDR vs non-MDR TB	ν	0.44 (solved)	
Endogenous reactivation rate, per year	$\upsilon_{hiv=0}$	0.001	[56]
Endogenous reactivation rate for HIV positive, per year	$\upsilon_{hiv=1}$	0.023	[56]
Proportion of infections progressing rapidly to active TB	$\phi_{hiv=0}$	0.14	[52]
Proportion of infections progressing rapidly to active TB in HIV infected individuals	$\phi_{hiv=1}$	0.4	[53]
Relative protection from reinfection in latent/recovered TB in HIV negative individuals	$\sigma_{hiv=0}$	0.6	[52]
Relative protection from reinfection in latent/recovered TB in HIV positive individuals	$\sigma_{hiv=1}$	0	(a)
Reduced mortality in smear negative compared to smear positive cases (HIV negative only)	ψ	0.29	[48]
Baseline adult mortality rate, per year	$\mu_{hiv=0}$	1/50	[46]
Adult mortality rate in HIV infected individuals, per year	$\mu_{hiv=1}$	1/20	[47]
TB mortality rate in smear positive cases, per year	$\mu^{TB}_{hiv=0,sm=1}$	0.22	[48]
TB mortality rate in smear negative cases, per year	$\mu^{TB}_{hiv=0,sm=0}$	Derived (see detail)	
TB mortality rate in HIV infected individuals, per year	$\mu^{TB}_{hiv=1,sm=1}$, $\mu^{TB}_{hiv=1,sm=0}$	1	[50, 51]
Spontaneous cure rate for smear positive HIV uninfected individuals, per year	$\gamma_{hiv=0,sm=1}$	0.1	[48]
Spontaneous cure rate for smear negative HIV uninfected individuals, per year	$\gamma_{hiv=0,sm=0}$	0.27	[48]

Parameter	Symbol	Baseline	Source
Spontaneous cure rate for HIV infected individuals, per year	$\gamma_{hiv=1,sm=1}$, $\gamma_{hiv=1,sm=0}$	0	(a)
Duration of infectiousness before symptom onset, years	τ_{pre}	0.34 (solved)	
Duration of symptoms before healthcare seeking, years	τ_{symp}	0.4	[12]
Time between healthcare visits, years	τ_{visit}	0.16	[12]
Proportion of smear positive active TB	$\pi_{hiv=0}$	0.65	[17]
Proportion of smear positive active TB in HIV infected individuals	$\pi_{hiv=1}$	0.35	[17,45]
Rate of never diagnosed active TB cases seeking care at defined healthcare provider, per year	δ_{tcp}	Derived (see detail)	
Rate at which unsuccessfully diagnosed cases move between healthcare providers, per year	$\eta_{hcp.from,hcp.to}$	Derived (see detail)	
Proportion of never treated, smear positive and smear negative TB cases correctly diagnosed by informal providers	$\epsilon^*_{sm=1,par=0,hcp=inf}$ $\epsilon_{sm=0,par=0,hcp=inf}$	0	(a)
Proportion of never treated, smear positive TB cases correctly diagnosed by qualified private providers	$\epsilon^*_{sm=1,par=0,hcp=prv}$	0.38 (Solved)	
Proportion of never treated, smear positive TB cases correctly diagnosed by public providers	$\epsilon^*_{sm=1,par=0,hcp=prv}$	0.98	(a)
Proportion of never treated, smear negative TB cases correctly diagnosed by qualified private and public providers	$\epsilon^*_{sm=0,par=0,hcp=prv}$ $\epsilon^*_{sm=0,par=0,hcp=pub}$	0.2	(a)
Rate of correct diagnosis	$\epsilon_{sm,par,hcp}$	Derived (see detail)	
Proportion lost to follow up between positive diagnosis and initiation of treatment (smear positive)	$\alpha_{sm=1}$	0.15	[36]
Proportion lost to follow up between positive diagnosis and initiation of treatment (smear negative)	$\alpha_{sm=0}$	0.25	(a)
HIV infection rate, per year	ω	0.0039 (solved)	
Rate diagnosed TB cases put on treatment, per year	χ	Derived (see detail)	
Rate of movement between treatment regimens	ξ	Derived (see detail)	
Duration of first line treatment regimen, years	$\tau_{tx=fl}$	0.5	[1]
Duration of category 2 (retreatment) regimen, years	$\tau_{tx=rt}$	0.67	[1]

Parameter	Symbol	Baseline	Source
Duration of second line treatment regimen, years	$\tau_{tx=sl}$	1.67	[1]
Rate of treatment completion, per year	ρ	Derived (see detail)	
Proportion of treatments that are unsuccessful from first line and retreatment regimens in non-MDR cases, per year	$\theta_{tx=fl,mdr=0}$ $\theta_{tx=rt,mdr=0}$	Derived (see detail)	
Proportion of treatments that are unsuccessful from first line and retreatment regimens in MDR cases, per year	$\theta_{tx=fl,mdr=1}$ $\theta^{*}_{tx=rt,mdr=1}$	0.47	[49]
Proportion of treatments that are unsuccessful from second line regimen in MDR cases, per year	$\theta_{tx=sl,mdr=1}$	0.3	[34]
Rate of developing resistance while on treatment for drug susceptible-TB, per year	$\zeta_{mdr=0}$	0.008	[55]

(a) Model assumption
