# Supplementary material for: The Importance of Implementation Strategy in Scaling Up Xpert MTB/RIF for Diagnosis of Tuberculosis in the Indian Health-Care System: A Transmission Model
Source: PLoS Med. 2014 Jul 15;11(7):e1001674. doi: 10.1371/journal.pmed.1001674 (PMC4098913; doi:10.1371/journal.pmed.1001674)
Supplement: Text S1 — Details of derived parameters. (PDF) [file pmed.1001674.s005.pdf]

Text S1. Detail on derived parameters

Force of infection

Individuals can either be infected with MDR-TB (determined by $\lambda_{mdr=1}$) or non-MDR-TB (determined by $\lambda_{mdr=0}$) . The TB infection process assumes density dependence, with the force of infection dependent on the number of infectious individuals, the population size, and the transmission rate. Infectious individuals comprise infectious pre-symptomatic individuals (**I**), active TB but pre-diagnosis seeking individuals (**A**), diagnosis seeking but have not yet been diagnosed (**D**), those that have been successfully diagnosed and are awaiting treatment initiation (**Z**) and those on unsuccessful treatments ($\mathbf{T}_{succ=0}$). Transmissibility is reduced by a factor of κ in those with smear negative TB ($sm = 0$), those with pre-symptom development and those on unsuccessful treatment. In addition there is reduced transmissibility of MDR-TB by a factor of ν compared to those with non-MDR TB. **N** is the total size of the population. Treatment is assumed to render an individual immediately non-infectious.

The reduced fitness of MDR is modeled as a reduction in transmission rate, not as a change in the risk of progression to active TB. The reduced transmission probability was derived to give a stable epidemic with current rates of MDR-TB, and that it may therefore underestimate the true transmissibility of MDR-TB in settings where the proportion of MDR-TB is increasing over time.

$$\begin{aligned}
\lambda_{mdr=0} = \quad & \beta * \Bigg(\sum_i \sum_j \Big(\mathbf{A}_{sm=1,mdr=0,hiv=i,par=j} + \sum_k \Big(\mathbf{D}_{sm=1,mdr=0,hiv=i,par=j,hcp=k} \\
& + \mathbf{Z}_{sm=1,mdr=0,hiv=i,par=j,hcp=l} \Big) \Big) \\
& + \kappa * \sum_i \sum_j \Big(\sum_k \mathbf{I}_{sm=k,mdr=0,hiv=i,par=j} + \mathbf{A}_{sm=0,mdr=0,hiv=i,par=j} \\
& + \sum_k \Big(\mathbf{D}_{sm=0,mdr=0,hiv=i,par=j,hcp=k} + \mathbf{Z}_{sm=0,mdr=0,hiv=i,par=j,hcp=l} \Big) \Big) \Bigg) / \mathbf{N}
\end{aligned}$$

$$\lambda_{mdr=1} = \nu * \beta * \Bigg(\sum_i \sum_j \Big(\mathbf{A}_{sm=1,mdr=1,hiv=i,par=j} + \sum_k \Big(\mathbf{D}_{sm=1,mdr=1,hiv=i,par=j,hcp=k}$$

$$+ \mathbf{Z}_{sm=1,mdr=1,hiv=i,par=j,hcp=l}\Big)\Big)$$

$$+ \kappa * \sum_i \sum_j \Big(\sum_k \mathbf{I}_{sm=k,mdr=1,hiv=i,par=j} + \mathbf{A}_{sm=0,mdr=1,hiv=i,par=j}$$

$$+ \sum_k \Big(\mathbf{D}_{sm=0,mdr=1,hiv=i,par=j,hcp=k} + \mathbf{Z}_{sm=0,mdr=1,hiv=i,par=j,hcp=l}\Big)\Big)\Bigg)/\mathbf{N}$$

Mortality rates

The mortality rate of HIV negative, smear negative TB is lower than for smear positive TB. The mortality rate of HIV positive, smear negative TB is assumed to be the same as HIV positive, smear positive TB.

$$\mu^{TB}_{hiv=0,sm=0} = \psi * \mu^{TB}_{hiv=0,sm=1}$$

$$\mu^{TB}_{hiv=1,sm=0} = \mu^{TB}_{hiv=1,sm=1}$$

Rate of care seeking

We assume that individuals are less infectious prior to development of symptoms that prompt care seeking; we assumed that they were as infectious during this phase as individuals with less infectious TB. The length of this period (τ_{pre}) was fitted to overall disease duration (inferred from the prevalence:incidence ratio from WHO estimates). These individuals develop symptoms at a rate of $1/\tau_{pre}$.

Upon development of symptoms, individuals will seek care at either informal, qualified private or public healthcare providers. The rate at which they do this depends on the probability of approaching each type of healthcare provider (δ^*_{hcp}) and the time between symptom onset and diagnosis seeking (τ_{symp}).

The probability of approaching each healthcare provider is taken from *Kapoor et al.*[12]:

δ^*_{hcp}=

Informal	Private	Public
0.69	0.31	0

Therefore the rate of diagnosis seeking at healthcare provider i is equal to:

$$\delta_{hcp=i} = \delta^*_{hcp=i}/\tau_{symp}$$

If individuals are unsuccessfully diagnosed at one healthcare provider they will either return to the same healthcare provider or go to a different one after a delay ($\tau_{visit} = 2.0$ months). The probability of movement between healthcare providers and the length of the delay comes from *Kapoor et al.* [12]:

$\eta^*=$

	To Informal	To Private	To Public
From Informal	0.48	0.49	0.03
From Private	0.03	0.36	0.61
From Public	0	0	1

The rate of movement between healthcare providers is:

$$\eta_{hcp.from=i,hcp.to=j} = \eta^*_{hcp.from=i,hcp.to=j}/\tau_{visit}$$

Successful diagnosis

The proportion of cases that are successfully diagnosed leading to treatment initiation depends on the probability of the healthcare provider successfully diagnosing (ϵ^*) and the proportion of cases lost to follow up between positive diagnosis and initiation of treatment (α). α is greater for smear negative cases as diagnosis that is not based on smear usually takes a longer time and is therefore more likely to result in loss to follow-up before initiation of treatment. For first time infections:

$$\epsilon_{sm=i,par=0,hcp=j} = \epsilon^*_{sm=i,par=0,hcp=j} * (1 - \alpha_{sm=i})$$

For subsequent infections, we assume that as individuals have historically been diagnosed with TB, there will be an increase in the probability of diagnosis equal to half of the difference between 1 and the initial probability of diagnosis success:

$$\epsilon_{sm=i,par=1,hcp=j} = (1 - 0.5 * (1 - \epsilon^*_{sm=i,par=0,hcp=j})) * (1 - \alpha_{sm=i})$$

Rate of treatment initiation and completion

There is variability in the probability of successful diagnosis at different healthcare providers. We assume a half-month delay between successful diagnosis and treatment initiation. All diagnoses of first time infections initially result in first line therapy and all diagnoses of subsequent infections initially result in retreatment therapy (irrespective of MDR status). Therefore:

$$\chi_{par=0,mdr=1,tx=fl} = \chi_{par=0,mdr=0,tx=fl} = 1/(0.5/12) = 24$$

$$\chi_{par=1,mdr=1,tx=rt} = \chi_{par=1,mdr=0,tx=rt} = 1/(0.5/12) = 24$$

and $\chi = 0$ for other subscript combinations.

In addition, we assume that people with MDR-TB and with a history of TB treatment are switched from category 2 (i.e., inappropriate) therapy to second-line (appropriate) therapy based

on culture, which takes 3 months (i.e., rate of 12/3 = 4/year) to process. We assume that a small fraction of such individuals receive culture and have access to second-line therapy at any given time, and thus divide this rate by 5 to reflect such lack of access. This is controlled by the ξ parameter:

$$\xi_{par=1,mdr=1,tx1=rt,tx2=sl} = 0.2/(3/12) = 0.8$$

$$\xi = 0 \text{ for other subscript combinations.}$$

Treatment will not result in cure in all individuals. Individuals may alternatively fail treatment, default or die. Of those infected with non-MDR TB, we assume half of defaulters are still successfully cured as they have received enough treatment to sufficiently reduce the bacterial burden. No individuals with MDR are cured if they default. In addition no individuals who fail treatment are cured, irrespective of the infecting pathogen. All those that default without being cured or fail treatment, return to the active disease compartment ($\mathbf{A}$) after passing through a failed treatment compartment ($\mathbf{T_{succ=0}}$). These transitions are determined by θ:

$$\theta_{tx\neq sl,mdr=0} = 50\% * 0.06 + 100\% * 0.019 = 0.049$$

This represents the probability of unsuccessful treatment for HIV uninfected individuals with non-MDR TB. Calculated as the sum of half the probability of default (0.06) and the probability of failure (0.019) [1, 34].

The rate of treatment completion:

$$\rho_{tx=i} = 1/\tau_{tx=i}$$

The treatment success probabilities for first- and second-line treatment were taken from the 2012 Global Report [1]. Note that these probabilities are higher in our manuscript than in the actual publications, as we excluded deaths (which are counted separately in our model) and one-half of defaults (presumed to have self-cured) from the denominator.

MDR TB treatment

All cases with a history of TB treatment are initially placed on an 8-month retreatment regimen. We assume that the majority (70%) of individuals with MDR-TB will fail these therapies and remain infectious. A small proportion of failure cases are started on appropriate therapy (e.g., using culture-based drug susceptibility testing), with substantial delay in obtaining results (assuming second line therapy is available). The second line regimen requires longer treatment (20 months) and results in poorer treatment outcomes. Those failing any treatment regimen remain partially infectious until placed on appropriate treatment; those who default return to the active state, eventually re-seeking diagnosis and treatment (with a competing risk of TB death).

Impact of Xpert

Xpert is assumed to be available through a network of labs rather than at the point-of-care and takes the same amount of time as sputum smear for results delivery (e.g., for specimen transport and reporting of results) implying similar risks of loss to follow-up between diagnosis and treatment initiation. Where employed, the model assumes Xpert has the following effects:

	Description	Value	Parameter	Source
1	Sensitivity to smear negative TB	0.73	$\epsilon^*_{sm=0,par=0}$	[17]
2	Sensitivity to smear positive TB	0.98	$\epsilon^*_{sm=1,par=0}$	[17]
3	Smear negative lost to follow up	0.15	$\alpha_{sm=1}$	[36]
4	Treatment initiation for those diagnosed with MDR	$0.80^{(a)}$	$\chi_{mdr=1,tx=sl}$	[1]

(a) Calculated as 0.94 (the estimated sensitivity for RIF resistance) multiplied by (1-0.15) to adjust for those lost to follow up.
