# Supplementary material for: The Importance of Implementation Strategy in Scaling Up Xpert MTB/RIF for Diagnosis of Tuberculosis in the Indian Health-Care System: A Transmission Model
Source: PLoS Med. 2014 Jul 15;11(7):e1001674. doi: 10.1371/journal.pmed.1001674 (PMC4098913; doi:10.1371/journal.pmed.1001674)
Supplement: Text S2 — Model equations with supporting text. (PDF) [file pmed.1001674.s006.pdf]

Text S2. Model equations

Rates between compartments are determined by a system of ordinary differential equations, outlined below.

Equation 1

There are two uninfected compartments: for HIV positive and HIV negative individuals ($S_{hiv=1}$ and $S_{hiv=0}$). Individuals leave the uninfected compartment by becoming infected by either MDR-TB or non-MDR-TB. We used a closed population, so the number of individuals entering the uninfected compartment (i.e. population size reaching adulthood) is equal to the number of deaths in the other compartments. The *hiv* subscript indicates HIV status.

For HIV negative individuals:

$$
\begin{aligned}
d\mathbf{U}_{hiv=0}/dt = \quad & \sum_m \mu_{hiv=m} * \Bigg(\mathbf{U}_{hiv=m} + \sum_i \sum_j \mathbf{I}_{hiv=m,sm=i,mdr=j} + \sum_i \sum_j \sum_k \mathbf{A}_{hiv=m,sm=i,mdr=j,par=k} \\
& + \sum_i \sum_j \sum_k \sum_l \mathbf{D}_{hiv=m,sm=i,mdr=j,par=k,hcp=l} \\
& + \sum_i \sum_j \sum_k \sum_l \mathbf{Z}_{hiv=m,sm=i,mdr=j,par=k,hcp=l} \\
& + \sum_i \sum_j \mathbf{T}_{hiv=m,mdr=i,tx=j} + \sum_i \mathbf{R}_{hiv=m,mdr=i} \Bigg) \\
& + \sum_m \mu^{TB}_{hiv=m,sm=1} * \Bigg(\sum_{i=1} \sum_{j=1} \mathbf{A}_{hiv=m,sm=1,,mdr=i,par=j} \\
& + \sum_i \sum_j \sum_k \mathbf{D}_{hiv=m,sm=1,mdr=i,par=j,hcp=k} + \sum_i \sum_j \sum_k \mathbf{Z}_{hiv=m,sm=1,mdr=i,par=j,hcp=k} \Bigg) \\
& + \sum_m \mu^{TB}_{hiv=m,sm=0} * \Bigg(\sum_{i=1} \sum_{j=1} \mathbf{A}_{hiv=m,sm=0,mdr=i,par=j} \\
& + \sum_i \sum_j \sum_k \mathbf{D}_{hiv=m,sm=0,mdr=i,par=j,hcp=k} \\
& + \sum_i \sum_j \sum_k \mathbf{Z}_{hiv=m,sm=0,mdr=i,par=j,hcp=k} + \sum_i \sum_j \sum_k \mathbf{T}_{hiv=m,mdr=i,par=j,tx=k,succ=0} \Bigg) \\
& - \lambda_{mdr=1} * \mathbf{U}_{hiv=0} \\
& - \lambda_{mdr=0} * \mathbf{U}_{hiv=0} \\
& - \mu_{hiv=0} * \mathbf{U}_{hiv=0} \\
& - \omega * \mathbf{U}_{hiv=0}
\end{aligned}
$$

where μ is non-TB mortality, μ^{TB} is TB associated mortality, λ is the force of infection and ω is the rate of HIV infection.

For HIV positive individuals:

$$
\begin{aligned}
d\mathbf{U}_{hiv=1}/dt = \quad & \omega * \mathbf{U}_{hiv=0} \\
& - \mu_{hiv=1} * \mathbf{U}_{hiv=0} \\
& - \lambda_{mdr=1} * \mathbf{U}_{hiv=1} \\
& - \lambda_{mdr=0} * \mathbf{U}_{hiv=1}
\end{aligned}
$$

Equation 2

Latent compartments are divided by MDR and HIV status.

$$
\begin{aligned}
d\mathbf{L}_{hiv=x,mdr=y}/dt = \quad & [1 - \phi_{hiv=x}) * \lambda_{mdr=y} * \mathbf{U}_{hiv=x} \\
& + [1 - \phi_{hiv=x}) * \sigma_{hiv=x} * \lambda_{mdr=y} * \mathbf{L}_{hiv=x,mdr\neq y} \\
& + \gamma_{hiv=x,sm=1} * \Big(\mathbf{A}_{hiv=x,sm=1,par=0,mdr=y} \\
& + \sum_i \mathbf{D}_{hiv=x,sm=1,par=0,mdr=y,hcp=i} + \sum_i \mathbf{Z}_{hiv=x,sm=1,par=0,mdr=y,hcp=i}\Big) \\
& + \gamma_{hiv=x,sm=0} * \Big(\sum_i \mathbf{I}_{hiv=x,par=0,mdr=y,sm=i} + \mathbf{A}_{hiv=x,sm=0,par=0,mdr=y} \\
& + \sum_i \mathbf{D}_{hiv=x,sm=0,par=0,mdr=y,hcp=i} + \sum_i \mathbf{Z}_{hiv=x,sm=0,par=0,mdr=y,hcp=i}\Big) \\
& - \phi_{hiv=x} * \sigma_{hiv=x} * \lambda_{mdr=y} * \mathbf{L}_{hiv=x,mdr=y} \\
& - \sigma_{hiv=x} * \lambda_{mdr\neq y} * \mathbf{L}_{hiv=x,mdr=y} \\
& - \upsilon_{hiv=x} * \mathbf{L}_{hiv=x,mdr=y} \\
& - \mu_{hiv=x} * \mathbf{L}_{hiv=x,mdr=y} \\
& + \mathbf{1_{hiv}} * \omega * \mathbf{L}_{hiv=x,mdr=y}
\end{aligned}
$$

where ϕ is the proportion of cases progression rapidly to active TB, λ is the force of infection, σ is the protection factor from previous infection, γ is the spontaneous self cure rate, υ is the endogenous rate of activation, μ is the non-TB associated mortality. $\mathbf{1_{hiv}}$ is an indicator variable that is equal to -1 when $x = 0$ (i.e. for HIV uninfected) and equal to 1 when $x = 1$. ω is the rate of HIV infection.

The first line represents the infection of uninfected individuals of x HIV status with TB of y MDR

type resulting in non-rapid progression. The second line is the reinfection of non-y type latently infected individuals with y TB (i.e. the infection of MDR infected individuals with non-MDR TB or visa versa) resulting in non-rapid progression. The third and fourth lines represents the spontaneous self cure of primary infected individuals with smear positive TB. The fifth and sixth lines are the spontaneous self cure of primary infected individuals with smear negative TB. The seventh line is the new infection of latently infected individuals with y mdr status resulting in rapid progression. The eighth line is the new infection of latently infected individuals with non-y mdr status. The ninth line is the endogenous reactivation of latent TB. The tenth line is the underlying mortality of individuals with x HIV status. The final line is the HIV infection process.

Equation 3

Rate of change from the pre-symptomatic infectious compartment, divided by MDR status, HIV status, future smear status and infection parity.

For individuals with no treatment history ($par = 0$):

$$
\begin{aligned}
d\mathbf{I}_{hiv=x,sm=w,par=0,mdr=y}/dt = \quad & \pi_{sm=w,hiv=x} * \phi_{hiv=x} * \lambda_{mdr=y} * \mathbf{U}_{hiv=x} \\
& + \pi_{sm=w,hiv=x} * \phi_{hiv=x} * \sigma_{hiv=x} * \lambda_{mdr=y} * \mathbf{L}_{hiv=x,mdr=y} \\
& + \pi_{sm=w,hiv=x} * \phi_{hiv=x} * \sigma_{hiv=x} * \lambda_{mdr=y} * \mathbf{L}_{hiv=x,mdr\neq y} \\
& + \pi_{sm=w,hiv=x} * \upsilon_{hiv=x} * \mathbf{L}_{hiv=x,mdr=y} \\
& - \gamma_{sm=w,hiv=x} * \mathbf{I}_{hiv=x,sm=w,par=0,mdr=y} \\
& - \mathbf{I}_{hiv=x,sm=w,par=0,mdr=y}/\tau_{pre} \\
& - \mu_{hiv=x} * \mathbf{I}_{hiv=x,sm=w,par=0,mdr=y} \\
& + \mathbf{1_{hiv}} * \omega * \mathbf{I}_{hiv=x,sm=w,par=0,mdr=y}
\end{aligned}
$$

where π is the proportion of active TB cases that will progress to smear positive TB, ϕ is the proportion of cases progression rapidly to active TB, λ is the force of infection, σ is the protection factor from previous infection, υ is the endogenous activation of latent TB, γ is the spontaneous self cure rate, τ_{pre} is the time individuals are infectious pre symptoms, μ is the non-TB associated mortality.$\mathbf{1_{hiv}}$ is an indicator variable that is equal to -1 when $x = 0$ (i.e. for HIV uninfected) and equal to 1 when $x = 1$. ω is the rate of HIV infection.

The first line represents the rapid progressors of infected individuals. The second line is the latently infected individuals that get reinfected with the same MDR status and become fast progressors. The third line is the latently infected individuals that get reinfected with a different MDR status and become fast progressors. The fourth line represent latently infected individuals that have endogenous reactivation of TB. The fifth line are individuals that spontaneously self cure. The sixth line are those that progress to symptomatic active TB. The sixth line are those that die from

non-TB associated mortality. The final line is the HIV infection process.
For those with a history of treatment (treatment parity greater than one):

$$
\begin{aligned}
d\mathbf{I}_{hiv=x,sm=w,par=1,mdr=y}/dt = \quad & \pi_{sm=w,hiv=x} * \phi_{hiv=x} * \sigma_{hiv=x} * \lambda_{mdr=y} * \mathbf{R}_{hiv=x,mdr=y} \\
& + \pi_{sm=w,hiv=x} * \phi_{hiv=x} * \sigma_{hiv=x} * \lambda_{mdr=y} * \mathbf{R}_{hiv=x,mdr\neq y} \\
& + \pi_{sm=w,hiv=x} * \upsilon_{hiv=x} * \mathbf{R}_{hiv=x,mdr=y} \\
& - \gamma_{sm=w,hiv=x} * \mathbf{I}_{hiv=x,sm=w,par=1,mdr=y} \\
& - \mathbf{I}_{hiv=x,sm=w,par=0,mdr=y}/\tau_{pre} \\
& - \mu_{hiv=x} * \mathbf{I}_{hiv=x,sm=w,par=0,mdr=y} \\
& + \mathbf{1_{hiv}} * \omega * \mathbf{I}_{hiv=x,sm=w,par=0,mdr=y}
\end{aligned}
$$

The first line represent reinfected individuals (infected with $mdr = y$) that had previously been infected resulting in rapid progression. The second line is the same except for those infected with $mdr \neq y$. The third line represent previously infected individuals that undergo endogenous reactivation. The fourth line are individuals that spontaneously self cure. The fifth line are those that progress to symptomatic active TB. The sixth line are those that die from non-TB associated mortality. The final line is the HIV infection process.

Equation 4

Rate of change from the symptomatic active TB compartment, divided by MDR status, HIV status, smear status and infection parity.

For individuals with no treatment history ($par = 0$):

$$
\begin{aligned}
d\mathbf{A}_{hiv=x,sm=w,par=0,mdr=y}/dt \quad & = \mathbf{I}_{hiv=x,sm=w,par=0,mdr=y}/\tau_{pre} \\
& - \sum_i \delta_{par=0,hcp=i} * \mathbf{A}_{hiv=x,sm=w,par=0,mdr=y} \\
& - \gamma_{sm=w,hiv=x} * \mathbf{A}_{hiv=x,sm=w,par=0,mdr=y} \\
& - \mu_{hiv=x} * \mathbf{A}_{hiv=x,sm=w,par=0,mdr=y} \\
& - \mu^{TB}_{hiv=x,sm=w} * \mathbf{A}_{hiv=x,sm=w,par=0,mdr=y} \\
& + \mathbf{1_{hiv}} * \omega * \mathbf{A}_{hiv=x,sm=w,par=0,mdr=y}
\end{aligned}
$$

where τ_{pre} is the time individuals are infectious pre symptoms, δ is the rate of diagnosis seeking at the different health care providers, γ is the spontaneous self cure rate, μ is the non-TB associated mortality, μ^{TB} is the TB associated mortality. $\mathbf{1_{hiv}}$ is an indicator variable that is equal to -1 when $x = 0$ (i.e. for HIV uninfected) and equal to 1 when $x = 1$. ω is the rate of HIV infection.

The first line represents the progression of pre-symptomatic TB to symptomatic TB. The second line is the diagnosis seeking at the informal, qualified private and public sectors. The third line is the spontaneous self cure of TB. The fourth line is the non-TB associated deaths. The fifth line is the TB-associated deaths. The final line is the HIV infection process.

For subsequent infections (infection parity >1):

$$
\begin{aligned}
d\mathbf{A}_{hiv=x,sm=w,par=1,mdr=y}/dt \quad =& \mathbf{I}_{hiv=x,sm=w,par=1,mdr=y}/\tau_{pre} \\
&+ \pi_{sm=w,hiv=x} * \left(\sum_i \sum_j \rho_{tx=i} * \mathbf{T}_{hiv=x,par=j,mdr=y,tx=i,succ=0} \right) \\
&- \sum_i \delta_{par=1,hcp=i} * \mathbf{A}_{hiv=x,sm=w,par=1,mdr=y} \\
&- \gamma_{sm=w,hiv=x} * \mathbf{A}_{hiv=x,sm=w,par=1,mdr=y} \\
&- \mu_{hiv=x} * \mathbf{A}_{hiv=x,sm=w,par=1,mdr=y} \\
&- \mu^{TB}_{hiv=x,sm=w} * \mathbf{A}_{hiv=x,sm=w,par=1,mdr=y} \\
&+ \mathbf{1_{hiv}} * \omega * \mathbf{A}_{hiv=x,sm=w,par=1,mdr=y}
\end{aligned}
$$

where in addition to the above, ρ is the rate of treatment completion. Used here to represent the transfer of those that fail treatment $succ = 0$ back to active TB.

Equation 5

Rate of change from the diagnosis seeking compartment, divided by MDR status, HIV status, smear status, infection parity and healthcare provider.

$$
d\mathbf{D}_{hiv=x,sm=w,par=z,mdr=y,hcp=v}/dt =
$$

$$\mathbf{A}_{hiv=x,sm=w,par=z,mdr=y} * \delta_{par=z,hcp=v} * [1 - \epsilon_{sm=w,par=z,hcp=v})$$

$$+ \sum_i \mathbf{D}_{hiv=x,sm=w,par=z,mdr=y,hcp=i} * \eta_{hcp.from=i,hcp.to=v} * [1 - \epsilon_{sm=w,par=z,hcp=v})$$

$$- \sum_i \mathbf{D}_{hiv=x,sm=w,par=z,mdr=y,hcp=v} * \eta_{hcp.from=v,hcp.to=i}$$

$$- \gamma_{sm=w,hiv=x} * \mathbf{D}_{hiv=x,sm=w,par=z,mdr=y,hcp=v}$$

$$- \mu_{hiv=x} * \mathbf{D}_{hiv=x,sm=w,par=z,mdr=y,hcp=v}$$

$$- \mu^{TB}_{hiv=x,sm=w} * \mathbf{D}_{hiv=x,sm=w,par=z,mdr=y,hcp=v}$$

$$+ \mathbf{1_{hiv}} * \omega * \mathbf{D}_{hiv=x,sm=w,par=z,mdr=y}$$

where δ is the rate of diagnosis seeking at the different health care providers, η is the rate of movement between healthcare providers upon unsuccessful diagnosis, ϵ is the probability of successful diagnosis, γ is the spontaneous self cure rate, μ is the non-TB associated mortality. μ^{TB} is the TB associated mortality.$\mathbf{1_{hiv}}$ is an indicator variable that is equal to -1 when $x = 0$ (i.e. for HIV uninfected) and equal to 1 when $x = 1$. ω is the rate of HIV infection.

The first line represents the diagnosis attempt at healthcare provider v resulting in unsuccessful diagnosis. The second line is the influx of individuals that were unsuccessfully diagnosed at other healthcare providers, resulting in unsuccessful diagnosis at healthcare provider v. The third line is the movement to other healthcare providers following unsuccessful diagnosis. The fourth line is the spontaneous self cure of TB. The fifth line is the non-TB associated deaths. The sixth final line is the TB-associated deaths and the final line is the HIV infection process.

Equation 6

Rate of change from the successfully diagnosed awaiting treatment initiation compartment, separated by MDR status, HIV status, smear status, infection parity and healthcare provider.

$$d\mathbf{Z}_{hiv=x,sm=w,par=z,mdr=y,hcp=v}/dt =$$

$$\mathbf{A}_{hiv=x,sm=w,par=z,mdr=y} * \delta_{par=z,hcp=v} * \epsilon_{sm=w,par=z,hcp=v}$$

$$+ \sum_i \mathbf{D}_{hiv=x,sm=w,par=z,mdr=y,hcp=i} * \eta_{hcp.from=i,hcp.to=v} * \epsilon_{sm=w,par=z,hcp=v}$$

$$- \chi_{par=z,mdr=y,tx=fl} * \mathbf{Z}_{hiv=x,sm=w,par=z,mdr=y,hcp=v}$$

$$- \chi_{par=z,mdr=y,tx=rt} * \mathbf{Z}_{hiv=x,sm=w,par=z,mdr=y,hcp=v}$$

$$- \chi_{par=z,mdr=y,tx=sl} * \mathbf{Z}_{hiv=x,sm=w,par=z,mdr=y,hcp=v}$$

$$- \gamma_{sm=w,hiv=x} * \mathbf{Z}_{hiv=x,sm=w,par=z,mdr=y,hcp=v}$$

$$- \mu_{hiv=x} * \mathbf{Z}_{hiv=x,sm=w,par=z,mdr=y,hcp=v}$$

$$- \mu^{TB}_{hiv=x,sm=w} * \mathbf{Z}_{hiv=x,sm=w,par=z,mdr=y,hcp=v}$$

$$+ \mathbf{1_{hiv}} * \omega * \mathbf{Z}_{hiv=x,sm=w,par=z,mdr=y}$$

where δ is the rate of diagnosis seeking at the different health care providers, η is the rate of movement between healthcare providers upon unsuccessful diagnosis, ϵ is the probability of successful diagnosis, χ is the rate of treatment initiation by regimen type, γ is the spontaneous self cure rate, μ is the non-TB associated mortality, μ^{TB} is the TB associated mortality. $\mathbf{1_{hiv}}$ is an indicator variable that is equal to -1 when $x = 0$ (i.e. for HIV uninfected) and equal to 1 when $x = 1$. ω is the rate of HIV infection.

The first line represents the diagnosis attempt at healthcare provider v resulting in successful diagnosis. The second line is the influx of individuals that were unsuccessfully diagnosed at other healthcare providers, resulting in successful diagnosis at healthcare provider v. The third line is treatment initiation on first line (fl) regimen. The fourth line is the treatment initiation on retreatment (rt) regimen (only relevant where infection parity¿1). The fifth line is the treatment initiation on second line (sl) regimen (only relevant in MDR cases). The sixth line is the spontaneous self cure of TB. The seventh line is the non-TB associated deaths. The eighth line is the TB-associated deaths and the final line is the HIV infection process.

Equation 7

Rate of change from the treatment compartment, separated by MDR status, HIV status, infection parity, regimen type and whether the treatment was successful.

For those on treatment resulting in cure:

$$d\mathbf{T}_{hiv=x,par=z,mdr=y,tx=u,succ=1} =$$

$$\chi_{par=z,mdr=y,tx=u} * [1 - \theta_{mdr=y,tx=u}) * [1 - \zeta_{mdr=y}) * \sum_i \sum_j \mathbf{Z}_{hiv=x,par=z,mdr=y,hcp=i,sm=j}$$

$$+ \mathbf{1_{mdr}} * \Big(\sum_i \mathbf{T}_{hiv=x,par=z,mdr=y,tx=i,succ=1} * \xi_{par=z,mdr=y,tx.from=i,tx.to=u}\Big)$$

$$- \mathbf{1_{mdr}} * \Big(\sum_i \mathbf{T}_{hiv=x,par=z,mdr=y,tx=u,succ=1} * \xi_{par=z,mdr=y,tx.from=u,tx.to=i}\Big)$$

$$- \rho_{tx=u} * \mathbf{T}_{hiv=x,par=z,mdr=y,tx=u,succ=1}$$

$$- \mu_{hiv=x} * \mathbf{T}_{hiv=x,par=z,mdr=y,tx=u,succ=1}$$

$$+ \mathbf{1_{hiv}} * \omega * \mathbf{T}_{hiv=x,par=z,mdr=y,tx=u,succ=1}$$

where $\mathbf{1_{mdr}}$ is an indicator variable and equal to 1 when $y = 1$ and $z = 2$ and 0 otherwise. χ is the rate of treatment initiation by regimen type u, ξ is the rate of movement between regimen types, θ is the treatment failure proportion, ζ is the proportion of development of MDR in non-MDR TB cases (equal to 0 when $y = 1$), ρ is the rate of treatment completion, μ is the non-TB associated mortality rate.$\mathbf{1_{hiv}}$ is an indicator variable that is equal to -1 when $x = 0$ (i.e. for HIV uninfected) and equal to 1 when $x = 1$. ω is the rate of HIV infection.

The first line represents treatment initiation in those successfully diagnosed. The second and third lines are the movement between treatment regimens (equal to 0 except when MDR-TB cases get moved from first line to second line regimens - this represents the delay from culture results to detect drug sensitivity). The fourth line is treatment completion. The fifth line calculates the number of non-TB associated deaths and the final line calculates HIV infection.

For those on treatment resulting in failure or default:

$$d\mathbf{T}_{hiv=x,par=z,mdr=y,tx=u,succ=0} =$$

$$\chi_{par=z,mdr=y,tx=u} * \theta_{mdr=y,tx=u} * [1 - \zeta_{mdr=y}) * \sum_i \sum_j \mathbf{Z}_{hiv=x,par=z,mdr=y,hcp=i,sm=j}$$

$$+ \chi_{par=z,mdr=y,tx=u} * \zeta_{mdr=y} * \sum_i \sum_j \mathbf{Z}_{hiv=x,par=z,mdr=y,hcp=i,sm=j}$$

$$- \rho_{tx=u} * \mathbf{T}_{hiv=x,par=z,mdr=y,tx=u,succ=0}$$

$$- \mu_{hiv=x} * \mathbf{T}_{hiv=x,par=z,mdr=y,tx=u,succ=0}$$

$$- \mu^{TB}_{hiv=x,sm=0} * \mathbf{T}_{hiv=x,par=z,mdr=y,tx=u,succ=0}$$

$$+ \mathbf{1_{hiv}} * \omega * \mathbf{T}_{hiv=x,par=z,mdr=y,tx=u,succ=0}$$

The first line represents treatment initiation in those successfully diagnosed. The second line is the development of MDR for those initially infected with non-MDR TB. The third line is treatment completion. The fourth line calculates the number of non-TB associated deaths. The fifth line calculates the number of TB associated deaths (mortality assumed to be the same as for smear negative TB) and the final line calculates HIV infection.

Equation 8

Rate of change from the recovered compartment, separated by MDR status and HIV status.

$$
\begin{aligned}
d\mathbf{R}_{hiv=x,mdr=y} \quad &= \sum_i \sum_j \rho_{tx=j} * \mathbf{T}_{hiv=x,par=i,mdr=y,tx=j} \\
&+ [1 - \phi_{hiv=x}) * \sigma_{hiv=x} * \lambda_{mdr=y} * \mathbf{R}_{hiv=x,mdr\neq y} \\
&+ \gamma_{hiv=x,sm=1} * \Bigg(\mathbf{A}_{hiv=x,sm=1,par=1,mdr=y} \\
&+ \mathbf{D}_{hiv=x,sm=1,par=1,mdr=y} + \sum_i \mathbf{Z}_{hiv=x,sm=1,par=1,mdr=y,hcp=i} \Bigg) \\
&+ \gamma_{hiv=x,sm=0} * \Bigg(\sum_i \mathbf{I}_{hiv=x,sm=i,par=1,mdr=y} + \mathbf{A}_{hiv=x,sm=0,par=1,mdr=y} \\
&+ \mathbf{D}_{hiv=x,sm=0,par=1,mdr=y} + \sum_i \mathbf{Z}_{hiv=x,sm=0,par=1,mdr=y,hcp=i} \Bigg) \\
&- \phi_{hiv=x} * \sigma_{hiv=x} * \lambda_{mdr=y} * \mathbf{R}_{hiv=x,mdr=y} \\
&- \sigma_{hiv=x} * \lambda_{mdr\neq y} * \mathbf{R}_{hiv=x,mdr=y} \\
&- \upsilon_{x=i} * \mathbf{R}_{hiv=x,mdr=y} \\
&- \mu_{x=i} * \mathbf{R}_{hiv=x,mdr=y} \\
&+ \mathbf{1_{hiv}} * \omega * \mathbf{R}_{hiv=x,mdr=y}
\end{aligned}
$$

where ρ is the rate of treatment completion, ϕ is the proportion of cases progression rapidly to active TB, λ is the force of infection, σ is the protection factor from previous infection, γ is the spontaneous self cure rate, υ is the endogenous activation rate of latent TB and μ is the non-TB associated mortality rate. $\mathbf{1_{hiv}}$ is an indicator variable that is equal to -1 when $x = 0$ (i.e. for HIV uninfected) and equal to 1 when $x = 1$. ω is the rate of HIV infection.

The first line represents the completion of treatment resulting in cure. The second line represent the reinfection of previously infected individuals with the other MDR form of TB resulting in non-rapid progression to disease. The third and fourth lines are from the spontaneous self cure of smear positive TB. The fifth and sixth lines are from the spontaneous self cure of smear negative TB. The seventh line is the reinfection of individuals by y type of TB resulting in rapid progression to active disease. The eighth line is the reinfection of individuals by non-y type of TB. The ninth line is the endogenous reactivation of TB, the tenth line represent the deaths from non-TB associated mortality and the final line calculates HIV infection.
