# Supplementary material for: The Importance of Implementation Strategy in Scaling Up Xpert MTB/RIF for Diagnosis of Tuberculosis in the Indian Health-Care System: A Transmission Model
Source: PLoS Med. 2014 Jul 15;11(7):e1001674. doi: 10.1371/journal.pmed.1001674 (PMC4098913; doi:10.1371/journal.pmed.1001674)
Supplement: Text S3 — Sensitivity using gamma-distributed waiting time. (PDF) [file pmed.1001674.s007.pdf]

Text S3. Sensitivity using gamma distributed waiting time

Ordinary differential equation models implicitly assume exponentially distributed dwelling times within compartments. We conducted a sensitivity analysis where an additional active disease, pre-diagnosis seeking compartment was created (resulting in gamma-distributed times). We calculated the impact of Xpert across all rollout scenarios and compared it to the base model where there was only a single such compartment.

In all six different scenarios, the population level impact of Xpert was identical to the original model with exponential distributed dwelling times.
